# Engineering human pluripotent stem cell lines to evade xenogeneic transplantation barriers

**DOI:** 10.1016/j.stemcr.2023.12.003

**Published:** 2024-01-11

**Authors:** Hannah A. Pizzato, Paula Alonso-Guallart, James Woods, Jon P. Connelly, Todd A. Fehniger, John P. Atkinson, Shondra M. Pruett-Miller, Frederick J. Monsma, Deepta Bhattacharya

**Affiliations:** 1 Department of Immunobiology, University of Arizona College of Medicine, Tucson, AZ 85724, USA; 2 The New York Stem Cell Foundation Research Institute, New York, NY 10019, USA; 3 Department of Cell & Molecular Biology, St. Jude Children’s Research Hospital, Memphis, TN 38105, USA; 4 Center for Advanced Genome Engineering, St. Jude Children’s Research Hospital, Memphis, TN 38105, USA; 5 Division of Oncology, Department of Medicine, Washington University School of Medicine, St. Louis, MO 63110, USA; 6 Division of Rheumatology, Department of Medicine, Washington University School of Medicine, St. Louis, MO 63110, USA; 7 Department of Surgery, University of Arizona College of Medicine, Tucson, AZ 85724, USA; 8 BIO5 Institute, University of Arizona, Tucson, AZ 85724, USA

**Keywords:** human Pluripotent Stem Cells, Transplantation, rejection, T cells, natural killer cells, complement

## Abstract

Successful allogeneic human pluripotent stem cell (hPSC)-derived therapies must overcome immunological rejection by the recipient. To build reagents to define these barriers, we genetically ablated *β2M*, *TAP1*, *CIITA*, *CD74*, *MICA*, and *MICB* to limit expression of HLA-I, HLA-II, and natural killer (NK) cell activating ligands in hPSCs. Transplantation of these cells that also expressed covalent single chain trimers of Qa1 and H2-K^b^ to inhibit NK cells and CD55, Crry, and CD59 to inhibit complement deposition led to persistent teratomas in wild-type mice. Transplantation of HLA-deficient hPSCs into mice genetically deficient in complement and depleted of NK cells also led to persistent teratomas. Thus, T cell, NK cell, and complement evasion are necessary to prevent immunological rejection of hPSCs and their progeny. These cells and versions expressing human orthologs of immune evasion factors can be used to define cell type-specific immune barriers and conduct preclinical testing in immunocompetent mouse models.

## Highlights

• hPSCs were engineered for xenogeneic transplantation in immunocompetent mice
• hPSCs were devoid of HLA-I and -II as well as MICA and MICAB
• Inhibition of both NK cells and complement allowed graft persistence
• Inhibition of phagocytosis was not required for graft persistence

## Teaser

Bhattacharya and colleagues used genome editing to define immune barriers to hPSC transplantation. In their work, evasion of T cells, NK cells, and complement was necessary to overcome xenogeneic transplantation barriers in immunocompetent wild-type mice. These cells and reagents can be used to define immune barriers for hPSC-derived therapies in preclinical mouse and *in vitro* models.

## Introduction

Immunological rejection of pluripotent stem cell (PSC)-based therapies remains a limitation to their widespread adoption. To circumvent rejection, these therapies can be combined with systemic immunosuppression, but this increases the risk of infections and cancer (Adami et al., 2003; Fishman and Rubin, 1998). Alternatively, induced PSCs can be used autologously, but this has the practical limitation of scalability. A third option is genetic modification of the starting PSCs to evade immunological rejection. Indeed, many recent studies report PSC modifications that evade recognition by specific immune cells (Deuse et al., 2019, 2021; Han et al., 2019; Hu et al., 2023a; Rong et al., 2014; Xu et al., 2019; Yoshihara et al., 2020), yet the immunological barriers to transplantation vary between cell types and tissues. A resource of well-characterized PSCs edited to evade specific components of the immune system would, thus, be valuable to the community to define cell-type and organ-specific barriers to transplantation.

T cell-mediated rejection is the best understood immunological barrier to allografts due to mismatched human leukocyte antigen (HLA). Yet, while genetic removal of HLA would likely be necessary for the generation of universal donor PSCs, it is unlikely to be sufficient. Mice reject major histocompatibility complex (MHC)-deficient skin allografts almost as rapidly as transplants with intact mismatched MHC (Dierich et al., 1993; Grusby et al., 1993). As one mechanism, MHC-I normally engages natural killer (NK) cell inhibitory receptors (Storkus et al., 1989). In this setting, if activating receptors are also engaged by their cognate ligands, NK cells can kill donor cells. Still, NK cells alone cannot explain the rapid rejection of MHC-deficient mouse skin allografts, suggesting the contribution of other immune pathways. For example, graft-reactive antibodies and subsequent complement deposition also contribute to rejection (Feucht et al., 1991). Phagocytes also play a role in rejection, both by direct removal of donor cells and subsequent indirect priming of T cells to donor peptides (Hancock et al., 1983).

To create resources to define immunological barriers for specific cell types or organs, we have generated a series of genetic modifications of well-characterized H1 human embryonic stem cells (hESCs) (Thomson et al., 1998) engineered to evade immune recognition by the pathways discussed above. H1 cells devoid of HLA-I and -II and the NK cell activating ligands MHC class I chain-related proteins A and B (MICA and MICAB), and additionally expressing inhibitory proteins for NK cells, complement, and phagocytes can cross xenogeneic barriers and form teratomas in wild-type mice. By testing combinations of these immune evasion factors, we demonstrated that inhibition of both complement and NK cells are required for graft persistence, while inhibition of phagocytosis is dispensable. These results present a strategy for engineering universal human PSCs (hPSCs).

## Results

### Generation of an HLA-I/II and MICA/MICAB-deficient hESC line

**Figure 1:**
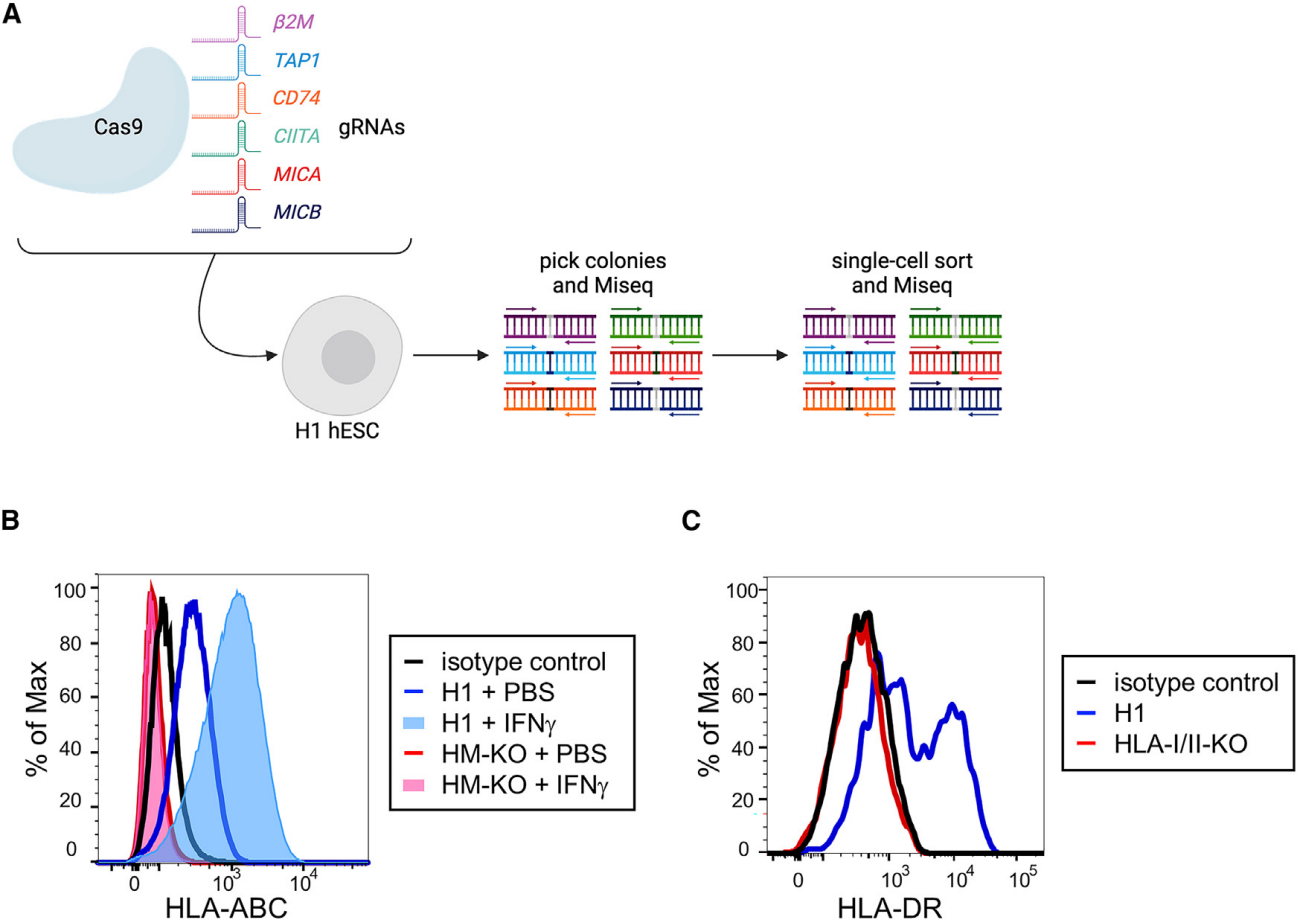
Generation of an HLA-I/II and MICA/B-deficient hESC line (A) Representation of the Cas9 editing strategy. Wild-type H1 hESCs were transfected with a Cas9 construct and up to three gRNAs at a time. gRNAs were for *β2M*, *TAP1*, *CD74*, *CIITA*, *MICA*, and *MICB*. Individual colonies were picked and sequenced to identify ones with frameshift mutations in target genes. Colonies with mutations were clonally sorted and re-sequenced. The resulting cells were then iteratively targeted by gRNAs to generate a line in which all alleles carried frameshift mutations. See also Figure S1 and Table S1. (B) Representative flow cytometric histograms of HLA-ABC (HLA-I) expression on H1 and HM-KO hESCs treated with PBS or IFNγ. (C) Representative histograms of HLA-DR (HLA-II) expression of DC-like cultures differentiated from H1 or HLA-I/II-KO hESCs.

We employed a CRISPR-Cas9 workflow to ablate six genes involved in T and NK cell recognition: *β2M* and *TAP1* to eliminate HLA-I and evade CD8^+^ T cells (Kaer et al., 1992; Koller et al., 1990; Zijlstra et al., 1990); *CD74* and *CIITA* to prevent HLA-II expression and CD4^+^ T cell recognition (Chang et al., 1996; Viville et al., 1993); and *MICA* and *MICB* to evade activating NKG2D receptors on NK cells (Bauer et al., 1999). Although individual mutations in *β2M* and *CIITA* likely eliminate all relevant HLA-I/II expression, prior studies in mice have suggested that residual MHC expression can still be observed in some knockout cell types (Bix and Raulet, 1992; Williams et al., 1998). Further ablation of *TAP1* and *CD74* would likely ensure the absence of surface HLA-I/II expression. H1 (wild-type) hESCs were iteratively nucleofected with a Cas9 construct and up to three guide RNA (gRNA)-encoding vectors to target each gene. The pool of transfectants was then subjected to MiSeq analysis to quantify the frequencies of frameshift mutations in each targeted gene. Individual colonies were picked and sequenced to identify those that carried frameshift mutations. Cells from these colonies were then single cell-sorted to generate clones. Another round of sequencing verified the mutations and confirmed the absence of mosaicism. Clones were then karyotyped. The overall workflow is shown in Figure 1A. Through this workflow, we generated a karyotypically normal hESC line carrying frameshift mutations in all alleles of *β2M*, *TAP1*, *CD74*, *CIITA*, *MICA*, and *MICB* (Table S1). For *MICA* and *MICB*, an additional round of targeting was required to ablate an in-frame and potentially functional fusion protein (Figure S1A). Through this process, we generated lines that are HLA-I/II-deficient (HLA-I/II-KO) and HLA-I/II and MICA/MICAB-deficient (HM-KO), summarized in Table S2.

Undifferentiated hESCs express very low levels of HLA-I and do not express HLA-II (Drukker et al., 2002). To confirm HLA-I deficiency, wild-type and HM-KO hESCs were treated with interferon γ (IFNγ) to induce HLA-I expression (Drukker et al., 2002). HM-KO cells lacked HLA-I expression, while unedited cells significantly upregulated expression upon IFNγ treatment (Figure 1B). To confirm ablation of HLA-II, hESCs were differentiated into activated dendritic cells (DCs). HLA-II was not detectably expressed by HLA-I/II-KO hESC-derived DC-like cells (Figure 1C).

Several recent reports showed CRISPR-Cas9 editing selects for cells with mutations in the proto-oncogene *TP53* (Haapaniemi et al., 2018; Ihry et al., 2018). After the first round of edits to generate the HLA-I-KO line, we indeed detected a CGG to CAG mutation in *TP53*, creating a missense R248Q change (Figure S1B), which is frequently observed in human cancers (Petitjean et al., 2007). No other potentially oncogenic or pathogenic sequence variants were observed by whole exome sequencing, as defined by the ClinVar database (Landrum et al., 2014). Fortuitously, the point mutation in *TP53* created a *de novo* PAM sequence and gRNA site, allowing for selective reversion of the sequence. Single-stranded donor oligonucleotides were designed such that the mutant CAG codon was reverted to AGG, which restores arginine, but can be distinguished from the wild-type allele at the nucleotide sequence (Figure S1B). These donor oligonucleotides were transfected into HM-KO hESCs with a gRNA-Cas9 RNA-protein complex. We screened by MiSeq and selected two clones that contained both AGG and CGG codons, functionally restoring the wild-type p53 amino acid sequence.

### Humanized NSG-W41 mice are incapable of rejecting allogeneic teratomas

**Figure 2:**
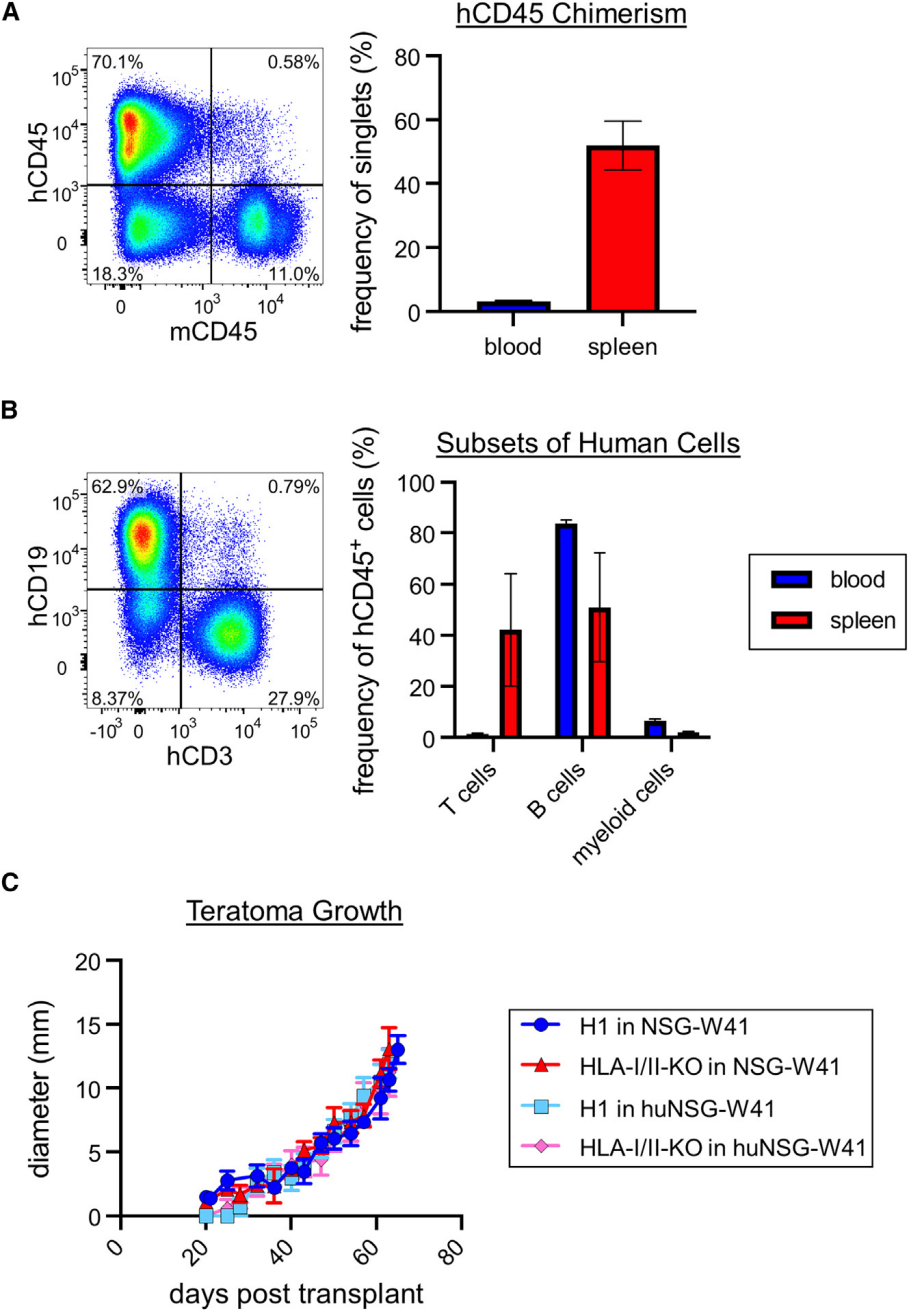
Humanized NSG-W41 mice are incapable of rejecting allogeneic teratomas (A) Donor chimerism of humanized NSG-W41 (huNSG-W41) mice. We transplanted 1 × 10^5^ human cord blood CD34^+^ cells into NSG-W41 mice. At ≥2 mo after transplant, mice were bled to confirm human chimerism (hCD45^+^), and a subset were sacrificed for splenic chimerism. A representative flow cytometry plot of splenic human chimerism gated on live cells (left). Blood and splenic chimerism are displayed as mean values ± SEM of 63 and 5 mice, respectively (right). (B) Cellular composition of human cells in huNSG-W41 mice. The human cells from blood and splenic samples were subgated on T cells (CD3^+^), B cells (CD19^+^), and myeloid cells (CD13^+^). A representative flow cytometry plot of splenic T and B cells gated on live human cells is shown (left). Mean values ± SEM are shown for blood (63 mice) and spleen (5 mice) (right). Chimerism values of individual mice are in Table S3. (C) Teratoma assay of H1 and HLA-I/II-KO hESCs in NSG-W41 and huNSG-W41 mice. We injected 1 × 10^6^ hESCs subcutaneously in the hind flank of a mouse. Mean values ± SEM are shown for each group of 5–10 mice from 2 independent experiments. p > 0.05 by two-way ANOVA with post hoc Tukey’s multiple comparisons test are not shown.

To test immune evasion *in vivo*, we generated humanized mice. NOD.Cg-*Kit*^*W−41J*^ *Tyr* ^+^ *Prkdc*^*scid*^ *Il2rg*^*tm1Wjl*^/ThomJ (NSG-W41) mice lack B, T, and NK cells and carry a mutation in *Kit* that allows robust, long-term engraftment of human HSCs without irradiation (Cosgun et al., 2014; McIntosh et al., 2015). We transplanted 1 × 10^5^ human cord blood CD34^+^ cells into NSG-W41 mice. At least 2 months post-transplant, we typically observed approximately 3% human chimerism in the blood and approximately 50% in the spleen, with robust B and T cell chimerism (Figures 2A and 2B, and Table S3), consistent with prior studies (Cosgun et al., 2014; McIntosh et al., 2015).

Using these humanized mice, we performed teratoma assays to test immune evasion by our cells. One million parental H1 or HLA-I/II-KO hESCs were embedded in Matrigel and injected subcutaneously in NSG-W41 or humanized NSG-W41 (huNSG-W41) mice. Unexpectedly, unmodified cells readily formed teratomas in humanized mice similarly to those in unhumanized mice (Figure 2C). We observed no negative correlations between the extent of human chimerism and teratoma growth. These data suggest these huNSG-W41 mice are not reliable representatives of human immune-mediated rejection. Though it is possible that other humanized systems might have performed better, each of these models has deficiencies ranging from MHC/HLA mismatches between thymic epithelial cells that educate T cells and peripheral antigen-presenting cells, lack of mouse cytokines binding human receptors, and graft versus host disease when mature T cells are transferred (Rongvaux et al., 2013).

### Immune evasion construct design and expression

Given the failure of huNSG-W41 mice to reject even wild-type human teratomas, we tested rejection in immunocompetent wild-type C57BL/6 (B6) mice. These animals have fully intact xenogeneic immune barriers, and, as such, represent an exceptionally high bar for transplantation (Brautbar et al., 1969). Thus, we considered it unlikely that HLA-I/II-KO hESCs would evade rejection in wild-type xenogeneic mice without further modifications. We first designed a series of constructs that encode mouse and human orthologs of immune evasion factors including CD46, CD55, and CD59 for complement (Liszewski et al., 1996); HLA-E and HLA-G for NK cells (Crew, 2007); and CD47 for phagocytosis (Oldenborg et al., 2000), as summarized in Table S4. Mice have orthologs of CD47, CD55, and CD59, but Crry in mice is the functional homologue of human CD46 (Holers et al., 1992). The mouse ortholog for HLA-E is the nonclassical MHC molecule Qa1 (Vance et al., 1998). There is no mouse ortholog for HLA-G, but H2-K^b^ inhibits Ly49C^+^ NK cells (Yu et al., 1996), which are unique to mice. MHC and HLA molecules can be covalently linked to a peptide and β2m to form single chain trimers (SCTs) (Crew et al., 2005; Yu et al., 2002). These trimers cannot exchange their peptide or β2m with endogenous HLA (Wang et al., 2009), and thus cannot rescue HLA deficiencies.

**Figure 3:**
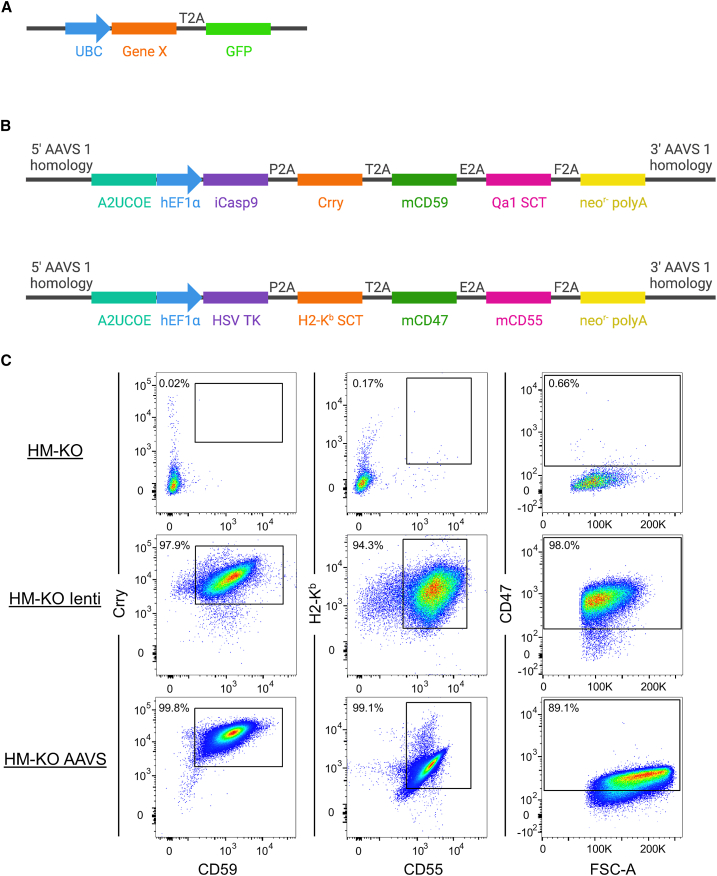
Mouse immune evasion construct design and expression (A) An example lentiviral construct for expression of immune evasion genes. Constructs contain a UBC promoter and GFP linked to an upstream immune evasion Gene X by a T2A sequence. (B) Two AAVS constructs encode mouse immune evasion genes. Each construct contains an A2UCOE insulator element and human elongation factor 1α (hEF1α) promoter. The immune evasion genes Crry, mCD59, Qa1 SCT, H2-K^b^ SCT, mCD47, and mCD55 are linked by 2A sequences. Each construct also has a suicide gene (iCasp9 or HSV TK), neomycin resistance gene, and 5′ and 3′ AAVS1 targeting homology arms. (C) Flow cytometric analyses of mouse inhibitory protein expression on HM-KO cells, as well as HM-KO cells transduced with all the mouse lentiviruses (HM-KO lenti) or transfected with both mouse AAVS constructs (HM-KO AAVS). See also Figures S2 and S3, and Table S4.

We designed two different types of constructs to test these immune evasion genes. We generated lentiviral constructs (Figure 3A), which allow for testing of HM-KO derivatives that express different combinations of factors. Each lentiviral construct encodes for a single mouse immune evasion factor linked by a T2A sequence to green fluorescent protein (GFP), with expression driven by the ubiquitin (UBC) promoter. We also designed adeno-associated virus site 1 (AAVS1) constructs encoding either mouse or human immune evasion proteins (Figures 3B and S2A, respectively) that target a specific integration site in the PPP1R12C locus (Sadelain et al., 2011). Each construct also contains an inducible suicide gene (herpes simplex virus [HSV], thymidine kinase [TK], or inducible caspase 9 [iCasp9]), which can be used to eliminate engrafted cells (upon ganciclovir or AP1903 treatment, respectively) (Hamel et al., 1996; Xie et al., 2001), and a neomycin resistance gene. Expression is driven by the human elongation factor 1α promoter, and genes are linked by 2A sequences (Donnelly et al., 2001).

We transduced HM-KO hESCS with either all the lentiviruses encoding mouse orthologs of immune evasion factors (HM-KO lenti) or transfected them with the AAVS constructs encoding either the mouse (HM-KO AAVS) or human (HM-KO hAAVS) factors. We confirmed protein expression and purified cells expressing all the proteins using fluorescence-activated cell sorting (FACS) (Figures 3C and S2B). HM-KO lenti cells had slightly higher protein expression than HM-KO AAVS cells.

Lentiviral transduction led to only modest silencing observed over time with passages. Yet for our AAVS-targeting constructs, despite multiple rounds of or constant drug selection and both bulk and clonal sorting, we repeatedly observed silencing (Figure S3A). Most AAVS1 constructs target a region downstream of the actual AAV integration site, which can become highly methylated and is, thus, prone to silencing (Ordovás et al., 2015). Therefore, we generated new immune evasion constructs and gRNAs to target the construct to the correct AAV integration site. We also included an A2UCOE insulator to further help prevent silencing (Zhang et al., 2007). These constructs allowed for the stable expression of the immune evasion factors over time (Figure S3B).

### HM-KO lenti and AAVS hESCs evade immune rejection in wild-type mice

**Figure 4:**
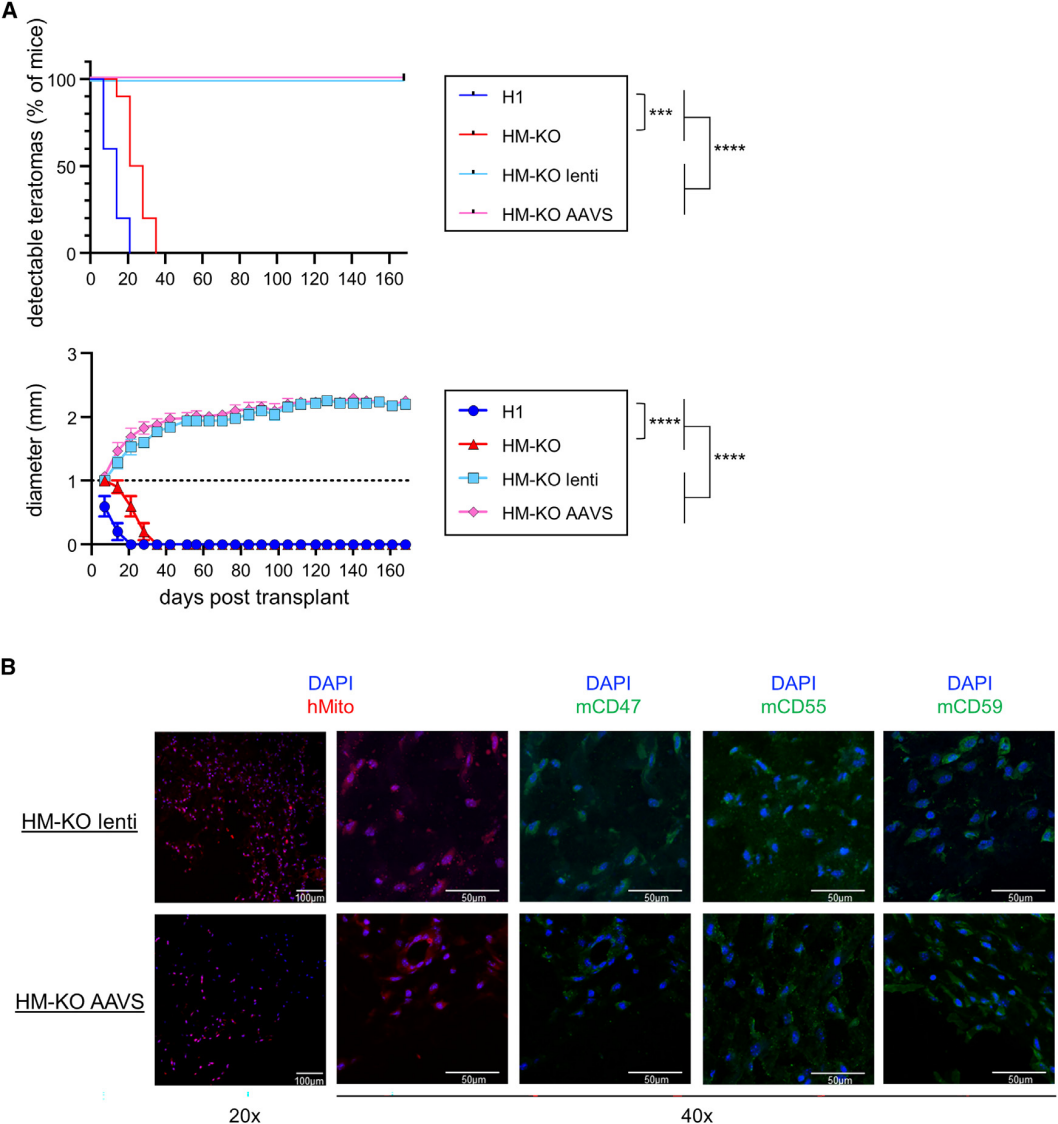
HM-KO lenti and AAVS hESCs evade immune rejection in wild-type mice (A) Teratoma assay of H1, HM-KO, HM-KO lenti, and HM-KO AAVS cells in B6 mice. Teratomas ≥1 mm in diameter were scored as detectable (top) and were measured (bottom). The dashed line at 1mm represents the limit of detection. Mean values ± SEM are shown for each group of 10 mice from 2 independent experiments. ^∗∗∗^p < 0.001 and ^∗∗∗∗^p < 0.0001 by log rank test for detection and two-way ANOVA with post hoc Tukey’s multiple comparisons test for growth. (B) Representative immunohistochemistry of sections of HM-KO lenti and AAVS teratomas removed 102 days after transplant in B6 mice. Sections were imaged at 20× magnification with 100-μm scale bars and ×40 with 50-μm scale bars. Sections were stained with DAPI and antibodies for human mitochondria (hMito) or mouse inhibitory proteins. See also Figures S4 and S5.

To quantify the ability of these edited cells to evade rejection, we performed teratoma assays in B6 mice using parental H1, HM-KO, HM-KO lenti, and HM-KO AAVS hESCs, with both the HM-KO lenti and AAVS lines expressing mouse proteins. While HM-KO teratomas persisted slightly longer than H1 teratomas, both were ultimately rejected (H1s within 3 weeks, and HM-KOs within 6 weeks) (Figure 4A). Encouragingly, both HM-KO lenti and AAVS teratomas persisted for at least 24 weeks in immunocompetent, xenogeneic recipients. The HM-KO lenti and AAVS lines grew and persisted similarly *in vivo*, suggesting that the gene delivery system and levels of overexpression did not affect immune evasion. HM-KO lenti and AAVS teratomas were harvested 102 days after transplantation, sectioned, and stained for human mitochondria and the mouse immune evasion factors, confirming the teratomas were of human origin and maintained inhibitory protein expression (Figure 4B). Nonetheless, while the engrafted HM-KO lenti and AAVS cells persisted, teratoma growth was stalled at approximately 2 mm in diameter (Figure 4A).

Given the plateauing of teratoma growth, we explored whether additional immune barriers remain. We treated mice with CD4, CD8, CD20, or NK1.1 depleting antibodies prior to transplanting hESCs and weekly throughout the duration of the assay. Efficient cell depletion of CD4^+^ T, CD8^+^ T, B, and NK cells was achieved within 4 days of the first antibody injection (Figure S4A). Yet none of these depletions further promoted the growth of HM-KO p53_mut_ lenti teratomas compared to controls (Figure S4B). These data suggest that immune lineage-derived factors may directly or indirectly lead to the differentiation, rather than rejection, of transplanted hESCs *in vivo*, halting teratoma growth.

We validated the human orthologs of these immune evasion genes and constructs *in vitro.* Undifferentiated hESCs express high endogenous levels of complement inhibitors and are resistant to lysis (Dormeyer et al., 2008). Therefore, to validate the human AAVS constructs for inhibition of complement, we utilized Chinese hamster ovary (CHO) cells. Expression of hCD55 and hCD46 on CHO cells decreased deposition of complement fragments C3c, C3d, and C4c relative to cells in the same cultures that did not express these proteins (Figures S5A–S5C). The isolated effects of CD59 were more difficult to assess, as it inhibits steps downstream of CD55 and CD46 (Liszewski et al., 1996).

Undifferentiated hESCs are also resistant to NK cell recognition and lysis (Drukker et al., 2002). Therefore, we utilized K562 cells, which are highly susceptible to NK cells, to test the inhibitory proteins encoded by our constructs. We verified protein expression on K562s (Figure S5D) and sorted cells that stably expressed these proteins. We incubated unmodified K562s or stably transfected K562s with human peripheral blood mononuclear cells. While unmodified K562s triggered robust NK cell degranulation, measured by CD107a surface expression, HLA-E^+^ K562s specifically inhibited NKG2A^+^ NK cell degranulation (Figures S5E and S5F). These assays demonstrate the proteins encoded by the human constructs are functional.

### Inhibition of both complement and NK cells is necessary to prevent immunological rejection

We next performed experiments to define which modifications were necessary and sufficient to evade rejection. Using lentiviruses, we generated lines expressing only the mouse complement, NK cell, or phagocytosis inhibitory factors described above. In addition, we made lentiviral constructs for mouse programmed death-ligand 1 (PD-L1) and CD24, which have been shown to inhibit phagocytosis (Barkal et al., 2019; Gordon et al., 2017), and transduced them into HM-KO cells (Figure S6). We also generated an AAVS construct of the complement inhibitor complement receptor 1 (CR1), due to its large insert size and poor packaging in lentiviruses (Figure S6).

**Figure 5:**
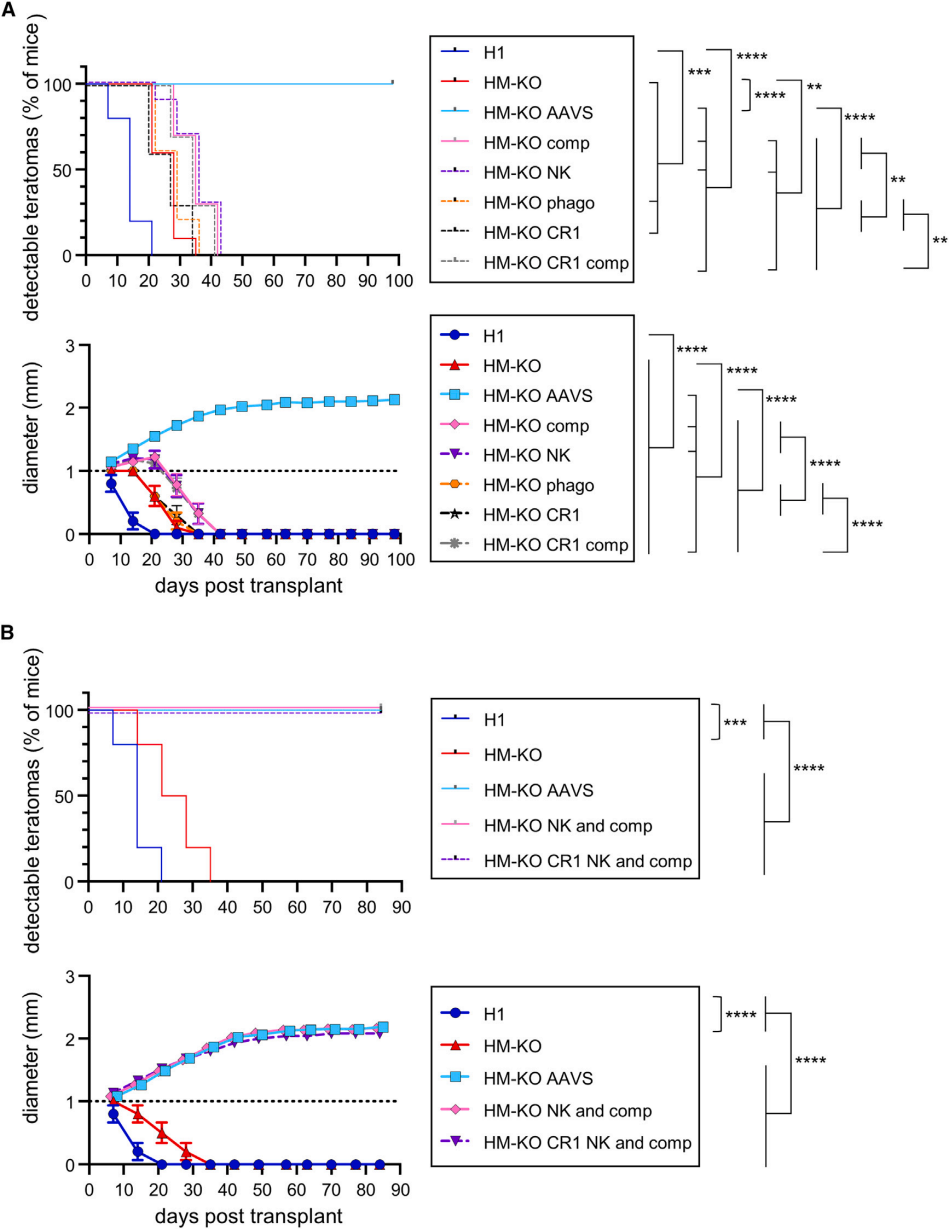
Inhibition of both complement and NK cells is necessary to prevent immunological rejection Teratoma assay of (A) H1, HM-KO, HM-KO AAVS, as well as HM-KO cells expressing individual groups of immune evasion factors and of (B) H1, HM-KO, HM-KO AAVS, as well as HM-KO cells expressing both NK and complement inhibitory proteins (lines outlined in Table S2) in B6 mice. Teratomas ≥1 mm in diameter were scored as detectable (top) and were measured (bottom). The dashed line at 1mm represents the limit of detection. Mean values ± SEM are shown for each group of 10 mice from 2 independent experiments. ^∗∗^p < 0.01, ^∗∗∗^p < 0.001, and ^∗∗∗∗^p < 0.0001 by log rank test for detection and two-way ANOVA with post hoc Tukey’s multiple comparisons test for growth. See also Figure S6.

Teratoma assays were performed on all the lines inhibiting individual pathways (Table S2) to determine which could prevent rejection similarly to the HM-KO AAVS line. None of these new lines formed teratomas akin to the HM-KO AAVS line (Figure 5A). However, relative to HM-KO teratomas, HM-KO comp, NK, and CR1 comp teratomas each persisted for longer and grew larger. HM-KO phago and CR1 teratomas did not provide any significant additional evasion relative to HM-KO teratomas (Figure 5A). We next tested whether the combined inhibition of NK cells and complement would achieve HM-KO AAVS levels of evasion. Indeed, HM-KO cells expressing inhibitory proteins for both NK cells and complement mirrored HM-KO AAVS teratomas (Figure 5B). Both the HM-KO comp and CR1 comp, as well as the HM-KO NK and comp and CR1 NK and comp, had similar persistence and growth, suggesting CR1 can replace Crry and CD55. HM-KO CR1 comp teratomas persisted longer and grew larger than HM-KO CR1 teratomas (Figure 5A), suggesting that CD59 provides additional protection. Overexpression of the phagocytosis inhibitors CD47, PD-L1, and CD24 was neither necessary nor sufficient to mediate immune evasion (Figures 5A and 5B). These results suggest that both complement and NK cells are major drivers of rejection in our system, and their inhibition is necessary for the persistence of teratomas.

### HM-KO cells persist in mice deficient in complement and NK cells

**Figure 6:**
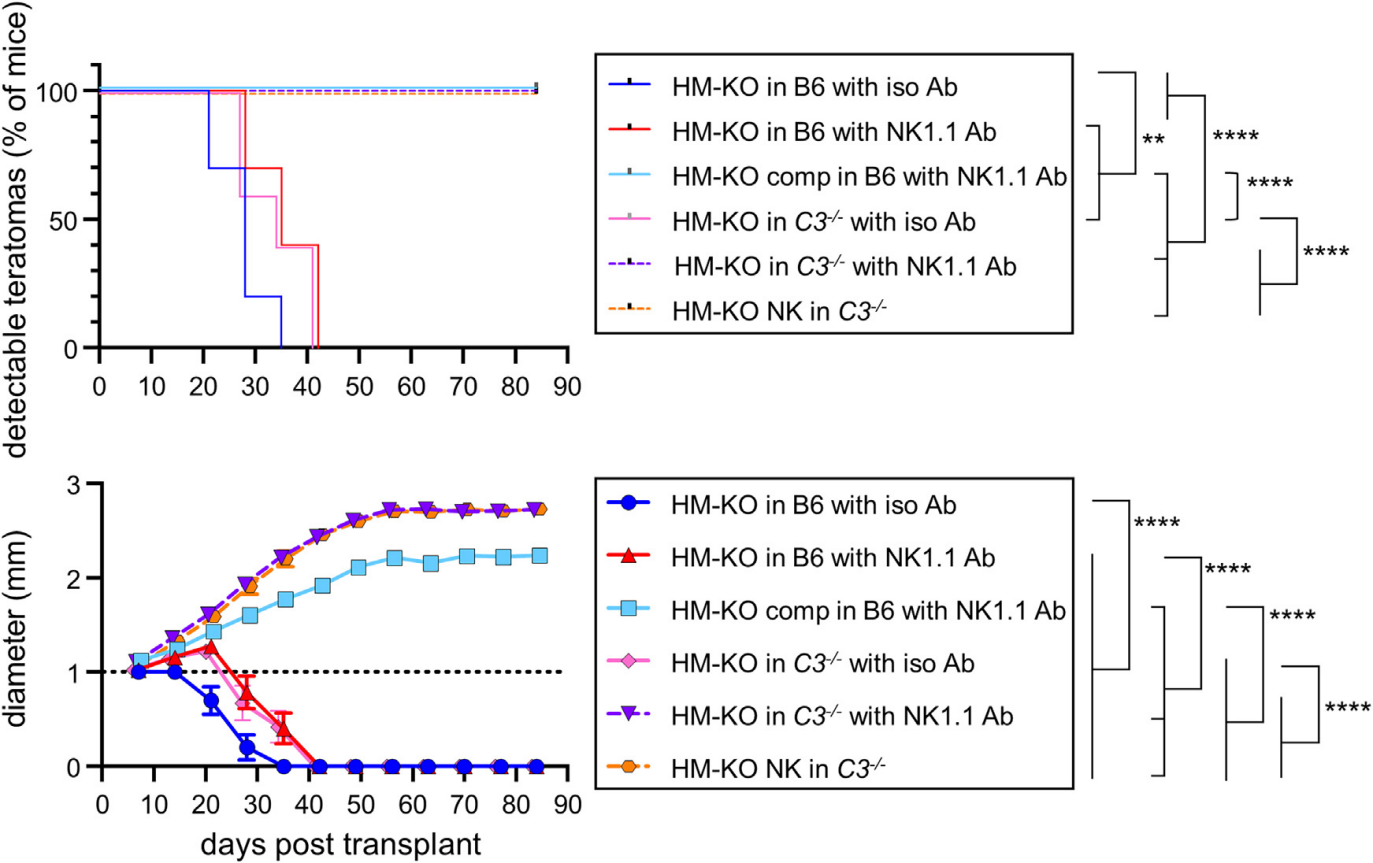
HM-KO cells persist in mice deficient in complement and NK cells B6 or *C3*^*−/−*^ mice were treated with an isotype control or an NK1.1-depleting antibody. Four days after antibody administration, a teratoma assay of HM-KO, HM-KO comp, or HM-KO NK cells was setup. Throughout the experiment, mice were given antibodies weekly. Teratomas ≥1 mm in diameter were scored as detectable (top) and were measured (bottom). The dashed line at 1mm represents the limit of detection. Mean values ± SEM are shown for each group of 10 mice from 2 independent experiments. ^∗∗^p < 0.01 and ^∗∗∗∗^p < 0.0001 by log rank test for detection and two-way ANOVA with post hoc Tukey’s multiple comparisons test for growth.

The immune evasion genes used above might potentially impact more than the known target pathways. To further investigate the importance of inhibiting both complement and NK cells, we measured teratomas using HM-KO cells in complement-deficient *C3*^*−/−*^ mice (Wessels et al., 1995) with or without NK cell depletion. NK cells were depleted using an NK1.1-depleting antibody prior to transplantation of HM-KO cells (Figure S4A) and weekly throughout the assay. Consistent with HM-KO NK and comp teratomas, both HM-KO teratomas in NK cell-deficient *C3*^*−/−*^ mice and HM-KO NK teratomas in *C3*^*−/−*^ mice persisted for at least 12 weeks after transplant (Figure 6). While these teratomas still plateaued in diameter, the size was slightly greater than seen with HM-KO AAVS and lenti teratomas in wild-type mice (Figures 4A and 6). HM-KO teratomas in NK cell-deficient *C3*^*−/−*^ mice and HM-KO NK teratomas in *C3*^*−/−*^ mice also grew larger than HM-KO comp teratomas in NK cell-deficient B6 mice (Figure 6). Given teratomas still plateau in *C3*^*−/−*^ mice, ablation of the complement pathways is not limiting teratoma size. HM-KO teratomas in NK cell-deficient B6 or in isotype control-treated *C3*^*−/−*^ mice mirrored HM-KO NK teratomas and HM-KO comp teratomas in B6 mice (Figures 5A and 6). These results confirm HM-KO cells persist with the inhibition or ablation of both complement and NK cells.

### Edited cells retain trilineage differentiation potential

**Figure 7:**
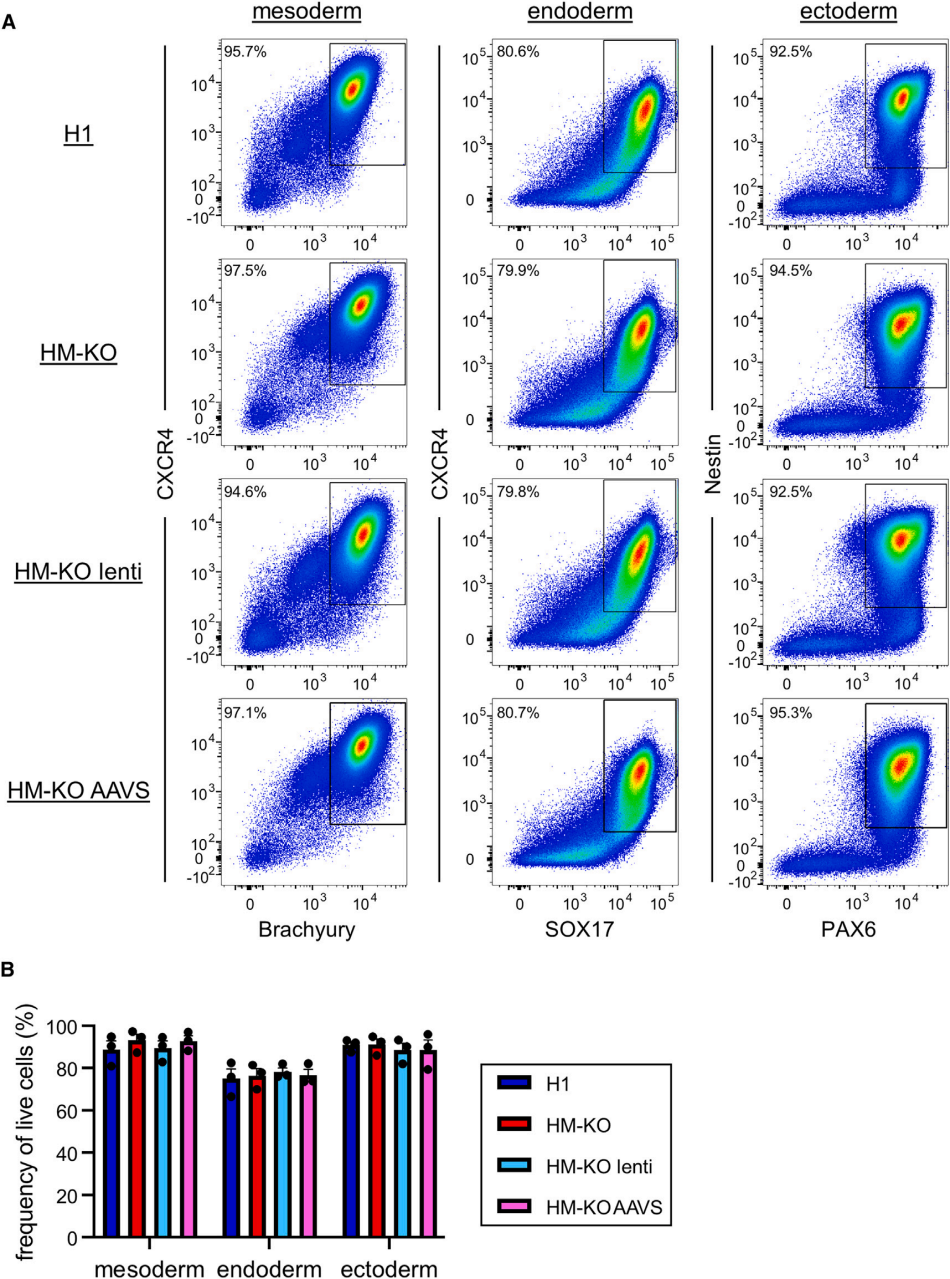
Edited cells retain trilineage differentiation potential (A) Representative flow cytometry plots of H1, HM-KO, HM-KO lenti, and HM-KO AAVS cells differentiated into mesoderm, endoderm, or ectoderm. (B) Frequency of live cells expressing markers for mesoderm (CXCR4^+^ Brachyury^+^), endoderm (CXCR4^+^ SOX17^+^), or ectoderm (Nestin^+^ PAX6^+^). Mean values ± SEM are shown from three independent experiments. p > 0.05 by two-way ANOVA with post hoc Tukey’s multiple comparisons test are not shown.

To determine whether these hESC edits affect pluripotency, we performed *in vitro* differentiations of H1, HM-KO, HM-KO lenti, and HM-KO AAVS cells. Each line was differentiated toward mesoderm, endoderm, and ectoderm lineages, then stained for markers of each germ layer, including CXCR4, BRACHYURY, SOX17, NESTIN, and PAX6. For each germ layer, all four lines demonstrated similar differentiation potential (Figures 7A and 7B), arguing against any substantial impact of these edits.

## Discussion

Immunological rejection represents the major barrier for transplants. This rejection is mediated by multiple arms of the immune system, including T cells, NK cells, phagocytes, and complement. Here, we described the generation of H1 hESCs ablated of HLA-I and -II, and MICA and MICAB to prevent T and NK cell-mediated recognition. Additionally, we designed constructs of both mouse and human inhibitory proteins for NK cells, phagocytes, and complement deposition. We demonstrated using teratoma assays that this line evades rejection and persists in immunocompetent mice. These modifications thus prevent xenorejection and may indicate barriers that must be overcome in allogeneic hPSC-based therapies. Aside from the obvious requirement that T cell recognition be ablated, we were able to pare down the important remaining modifications to those inhibiting NK cells and complement. These cells can thus serve as a starting point for stem cell researchers to define transplantation barriers and graft function in immunocompetent animals for cell types of interest.

Other recent studies have tested a variety of strategies to evade aspects of immunological rejection of PSCs and downstream cell types (Deuse et al., 2019, 2021; Gerace et al., 2023; Han et al., 2019; Hu et al., 2023a, 2023b; Rong et al., 2014; Xu et al., 2019; Yoshihara et al., 2020). Each of these studies incorporated inhibition or suppression of T cells as a requisite step, but additional immune evasion strategies, usually to evade NK cells (Gerace et al., 2023; Han et al., 2019; Xu et al., 2019), have differed. In one approach, ablation of HLA-I and -II with overexpression of CD47 was sufficient for PSCs and PSC-derived tissues to evade rejection (Deuse et al., 2019, 2021; Hu et al., 2023a, 2023b). Unexpectedly, PD-L1 overexpression on wild-type PSCs was also sufficient to allow engraftment (Yoshihara et al., 2020). Together, these studies suggest there may be multiple paths to evading rejection, but also highlight the lack of consensus standards to test immunogenicity of edited cells and grafts. The reported impact of CD47 overexpression is surprising, irrespective of the specific assay. Independent groups have found only modest impacts of CD47 blockade on NK cell activity or toxicity for non-malignant cell types other than aged red blood cells (Gerace et al., 2023; Sikic et al., 2019). Additionally, in our experiments we were unable to observe any impact of CD47 or PD-L1 on graft acceptance.

No single method to test immunogenicity is without drawbacks, and it is worth considering caveats to each. In our humanized mouse models, despite robust human B and T cell splenic engraftment, even unmodified hESCs were tolerated. Specific aspects of human responses can be tested *in vitro*, but it is difficult to accurately account for the complex cross-talk between immune cells and molecules that would occur in anatomically regulated ways *in vivo*. Another option is to test immune evasion using orthologous strategies in species-matched allogeneic transplants. While this is a strong approach, it does not directly test the modified hPSC products that would ultimately be used therapeutically. We, therefore, utilized xenotransplants as a readout for immunogenicity with the full acknowledgment of several caveats. For instance, many of the immune evasion proteins in our constructs have mouse and human orthologs, but some do not. However, crossing xenogeneic immune barriers is generally considered to be a higher bar than allogeneic transplantation, even when MHC is mismatched (Brautbar et al., 1969).

A limitation of our study is the exclusive use of teratomas to examine immunogenicity. PSCs and differentiated cells may have different immune barriers based on the intrinsic properties of the cell type and the anatomical and orthotopic site of transplantation. Nonetheless, that xenogeneic barriers can be overcome at all in any cell type through genetic modifications lends confidence that the development of universal hPSC-based therapies is possible. Moreover, hESC lines, based on well characterized H1 cells, which can cross mouse xenobarriers, can be incorporated rapidly into ongoing translational efforts by other investigators to test in their cells of interest.

## Experimental procedures

### Resource availability

#### Corresponding author

Further information and requests should be directed to Deepta Bhattacharya (deeptab@arizona.edu).

#### Materials availability

HM-KO, HM-KO hAAVS, HM-KO mAAVS, and HM-KO mlenti hESC cell lines are being deposited with WiCell and in the meantime will be available from the lead contact with a Materials Transfer Agreement and WiCell approval. Plasmids generated in this study are available from Addgene with the following names and plasmid IDs: human AAVS plasmids are AAVS1-A2UCOE-EF1a-hCD46-P2A-HLA-G SCT-T2A-hCD47-E2A-hCD59-F2A-neo-AAVS1 (205443) and AAVS1-A2UCOE-EF1a-iCasp9-P2A-HLA-E SCT-T2A-hCD55-E2A-neo-AAVS1 (205444); mouse AAVS plasmids are AAVS1-A2UCOE-EF1a-iCasp9-P2A-Crry-T2A-mCD59-E2A-Qa1 SCT-F2A-neo-AAVS1 (205445), AAVS1-A2UCOE-EF1a-HSV TK-P2A-H2-Kb SCT-T2A-mCD47-E2A-mCD55-F2A-neo-AAVS1 (205446), and AAVS1-A2UCOE-EF1a-mCR1-F2A-neo-AAVS1 (205447); mouse lentiviral plasmids are TRE-UBC- mCD24-T2A-GFP (205448), TRE-UBC-mCD47-T2A-GFP (205449), TRE-UBC-mCD55-T2A-GFP (205450), TRE-UBC-mCD59-T2A-GFP (205451), TRE-UBC-Crry-T2A-GFP (205452), TRE-UBC-Qa1 SCT-T2A-GFP (205453), TRE-UBC-H2-Kb SCT-T2A-GFP (205454), and TRE-UBC-mPD-L1-T2A-GFP (205455); human lentiviral plasmids are TRE-UBC-hCD24-T2A-GFP (205456), TRE-UBC-hCD46-T2A-GFP (205457), TRE-UBC-hCD47-T2A-GFP (205458), TRE-UBC-hCD55-T2A-GFP (205459), TRE-UBC-hCD59-T2A-GFP (205460), TRE-UBC-HLA-E SCT-T2A-GFP (205461), and TRE-UBC-HLA-G SCT-T2A-GFP (205462).

#### Data and code availability

Raw data from whole exome sequencing is available at the NCBI Sequence Read Archive (accession number SRA: PRJNA987027).

### hESC cell culture

WA01 (H1) hESCs were obtained from WiCell at passage 46 and expanded to generate a master bank within the first 5 passages. hESCs were cultured on Matrigel (Corning, cat. no. 354277) in mTeSR1 or mTeSR Plus (STEMCELL Technologies, cat. no. 85850 and 100-0276). Passaging was performed with Accutase (Innovative Cell Technologies, cat. no. AT104-500) at split ratios from 1:6 to 1:10. hESCs were thawed in mTeSR with 10μM Y-27632 (hydrochloride) ROCK inhibitor (Cayman Chemical Company, cat. no. 10005583) and were frozen in mFreSR (STEMCELL Technologies, cat. no. 05855). Following FACS, hESCs were cultured with CloneR (STEMCELL Technologies, cat. no. 05888) per manufacturer’s instructions. Cultures were maintained at 37°C with 5% CO_2_. Cells were kept in sterile conditions and monitored regularly for contamination or abnormalities.

### Generation of HM-KO hESCs

H1 hESCs were pretreated with Revitacell (Thermo Fisher Scientific, cat. no. A2644501) for 1h, then nucleofected (Lonza Nucleofector 4D, program CA-137) with a Cas9 construct (modified version of pMJ915, a gift from Chris Jeans) and up to three gRNA-encoding vectors to target each gene. The pool of transfectants was then subjected to MiSeq analysis (Illumina) to estimate the frequency of frameshift mutations in each targeted gene. Individual colonies were picked and screened via Miseq to identify clones with frameshift mutations, which were then single cell sorted. Another round of Miseq was performed to verify the mutations and confirm the absence of mosaicism. Clones were karyotyped by Cell Line Genetics. Clones with normal karyotypes were expanded and frozen down within the first five passages, which were used to generate subsequent lines. The gRNAs and primers used for each targeted gene are listed in Table S5. See the Supplemental experimental procedures for the *TP53* mutation correction and whole exome sequencing.

### Flow cytometry

Cells were resuspended in PBS with 5% adult bovine serum and 2 mM ethylenediaminetetra-acetic acid prior to staining. hESCs being sorted were harvested and stained in mTeSR Plus with CloneR. Transcription factor staining was performed with True-Nuclear Transcription Factor Buffer Set (BioLegend) per the manufacturer’s instructions. See the Supplemental experimental procedures for the antibodies used. Cells were analyzed on a BD LSR II, BD Fortessa, or Cytek Aurora. FACS was performed on a BD FACSAria II or III. Data were analyzed on FlowJo (FlowJo Enterprise). hESCs were treated with PBS or 20 ng/mL hIFNγ (PeproTech) for 24 h to induce HLA-I expression.

### Differentiations

STEMdiff Trilineage Differentiation Kit (STEMCELL Technologies, cat. no. 05230) was used for trilineage differentiations per the manufacturer’s instructions at middle and late passages. See Supplemental experimental procedures for DC-like cell differentiations.

### Mouse lines

All animal procedures were approved by the Animal Care and Use Committees at Washington University in St. Louis and the University of Arizona. NOD.Cg-*Kit*^*W−41J*^ *Tyr* ^+^ *Prkdc*^*scid*^ *Il2rg*^*tm1Wjl*^/ThomJ (NSG-W41) (McIntosh et al., 2015) and B6.129S4-*C3*^*tm1Crr*^/J (*C3*^*−/−*^) (Wessels et al., 1995) mice were obtained from The Jackson Laboratory (stock no. 026622 and 029661, respectively). C57BL6/N mice were obtained from Charles River Laboratories.

### Humanized mice

Human cord blood was obtained from St. Louis Cord Blood Bank. CD34^+^ cells were enriched with Dynabeads CD34 Positive Isolation Kit (Life Technologies, cat. no. 11301D) per the manufacturer’s instructions. We injected 1 × 10^5^ cells retro-orbitally into unconditioned NSG-W41 recipients. At least 2 months after transplant, mice were checked for human chimerism. See the Supplemental experimental procedures for blood and spleen processing.

### Teratoma assays

hESCs were harvested and resuspended at 10 × 10^6^ cells/mL in a 1:1 mix of Matrigel and PBS. We injected 100 μL subcutaneously into the hind flank of a mouse. Teratoma growth was monitored weekly and measured with a caliper once the diameter was confidently measurable (approximately 1.2 mm). See the Supplemental experimental procedures for teratoma immunohistochemistry.

### Lentivirus and AAVS construct design and transduction/transfection

Immune evasion genes were cloned without stop codons into lentiviral vectors downstream of a UBC promoter and in-frame with a downstream T2A-GFP cassette (base vector was a gift from A. Bredemeyer, Washington University) (Wang et al., 2014). Lentivirus production is detailed in the Supplemental experimental procedures. We used 5–10 μL lentivirus to transduce one well of hESCs in a six-well plate. Transduced cells were sorted for GFP and protein expression. AAVS constructs were gene-synthesized by BioBasic. AAVS constructs were transfected into hESCs along with LentiCRISPRv2-mCherry (Addgene 99154), encoding a gRNA for human AAVS1 targeting (GGAAGAGAGTAGGTCGAAG) using GeneJuice Transfection Reagent. Transfected cells were selected with 100 μg/mL neomycin and sorted.

### Statistics

All statistical analyses were conducted using GraphPad Prism and are detailed in each figure legend.

### Figures

Figures were created with BioRender.

## Supplemental information

Document S1. Figures S1–S6, Tables S1–S5, and Supplemental experimental procedures

Document S2. Article plus supplemental information
