# Engineering human pluripotent stem cell lines to evade xenogeneic transplantation barriers

**DOI:** 10.1016/j.stemcr.2024.03.006

**Published:** 2024-03-21

**Authors:** Hannah A. Pizzato, Paula Alonso-Guallart, James Woods, Bjarki Johannesson, Jon P. Connelly, Todd A. Fehniger, John P. Atkinson, Shondra M. Pruett-Miller, Frederick J. Monsma, Deepta Bhattacharya

## Main text

(Stem Cell Reports *19*, 299–313; February 13, 2024)

During revisions performed between an earlier, pre-print version of this article posted to *bioRxiv* and the version submitted to *Stem Cell Reports*, Bjarki Johannesson’s name was mistakenly omitted from the author list. Bjarki Johannesson now appears in the author list between James Woods and Jon P. Connelly, and the authors apologize for the oversight.

