# Supplementary material for: Engineering human pluripotent stem cell lines to evade xenogeneic transplantation barriers
Source: Stem Cell Reports. 2024 Jan 11;19(2):299–313. doi: 10.1016/j.stemcr.2023.12.003 (PMC10874864; doi:10.1016/j.stemcr.2023.12.003)
Supplement: Document S1. Figures S1–S6, Tables S1–S5, and Supplemental experimental procedures [file mmc1.pdf]

Stem Cell Reports, Volume *19*

Supplemental Information

Engineering human pluripotent stem cell lines to evade xenogeneic transplantation barriers

Hannah A. Pizzato, Paula Alonso-Guallart, James Woods, Jon P. Connelly, Todd A. Fehniger, John P. Atkinson, Shondra M. Pruett-Miller, Frederick J. Monsma Jr., and Deepta Bhattacharya

Figure S1

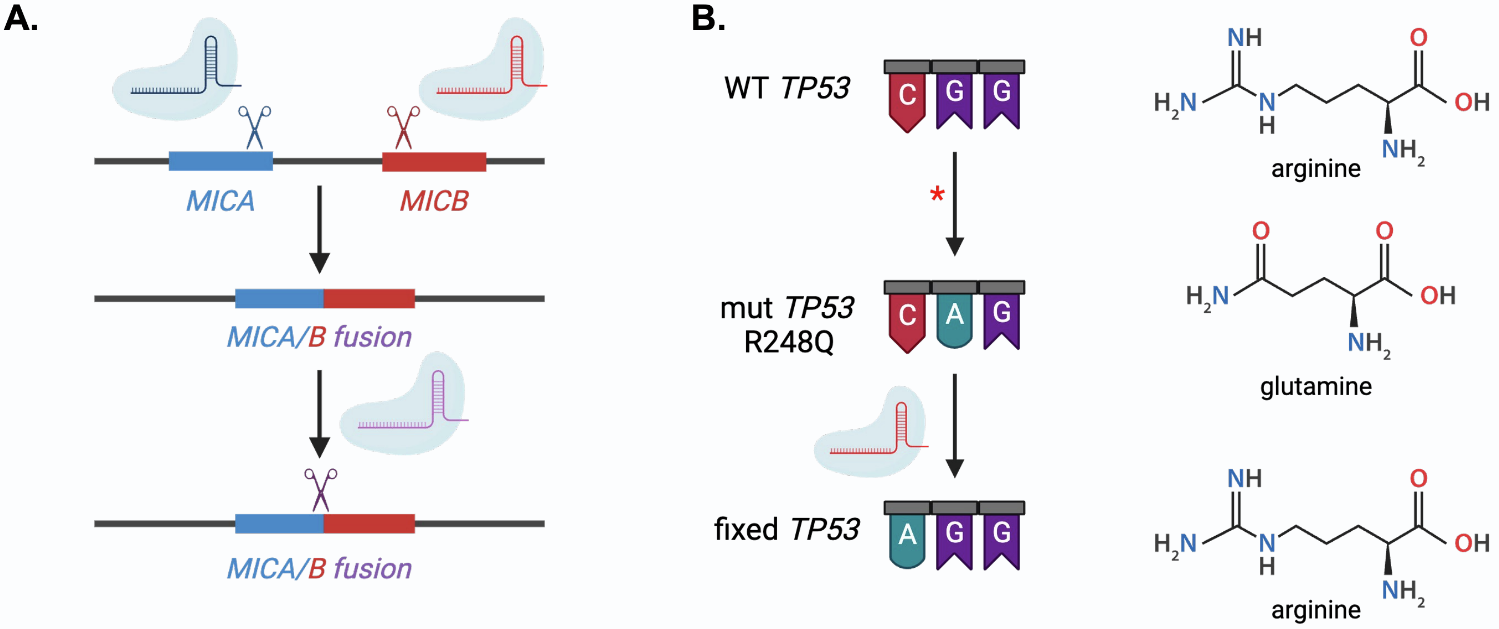
A.
MICA
MICB
MICA/B fusion
MICA/B fusion
B.
WT TP53
C G G
*
mut TP53
R248Q
C A G
fixed TP53
A G G
arginine
glutamine
arginine

Figure S1, related to Figure 1. Ablation of the MICA/MICB locus and correction of a p53 mutation in HLA-deficient hESCs. (A) Schematic of the *MICA/B* fusion generated in one allele. gRNAs for *MICA* and *MICB* cut their respective genes but left behind an in-frame fusion of *MICA* and *MICB* in one allele. This fusion was edited out of frame with a different gRNA. (B) Schematic of the R248 residue of wild-type *TP53*, the identified mutation, and the reverted line. Wild-type p53 encodes an arginine (CGG) at position 248. In the first step of generating the HM-KO line, clones were inadvertently selected that carried a mutated CAG encoding glutamine. Using a gRNA and Cas9, this codon was reverted to an arginine-encoding AGG sequence.

Figure S2

A.

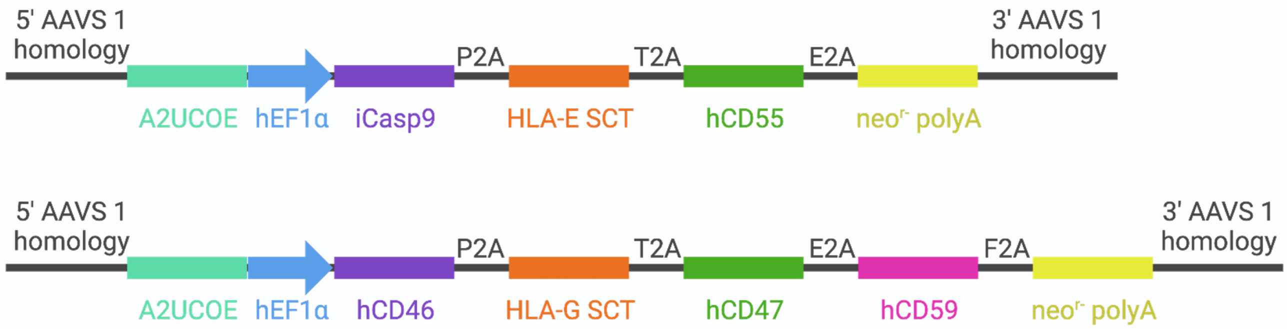
5' AAVS 1 homology
P2A
T2A
E2A
3' AAVS 1 homology
A2UCOE
hEF1α
iCasp9
HLA-E SCT
hCD55
neor- polyA
5' AAVS 1 homology
P2A
T2A
E2A
F2A
3' AAVS 1 homology
A2UCOE
hEF1α
hCD46
HLA-G SCT
hCD47
hCD59
neor- polyA

B.

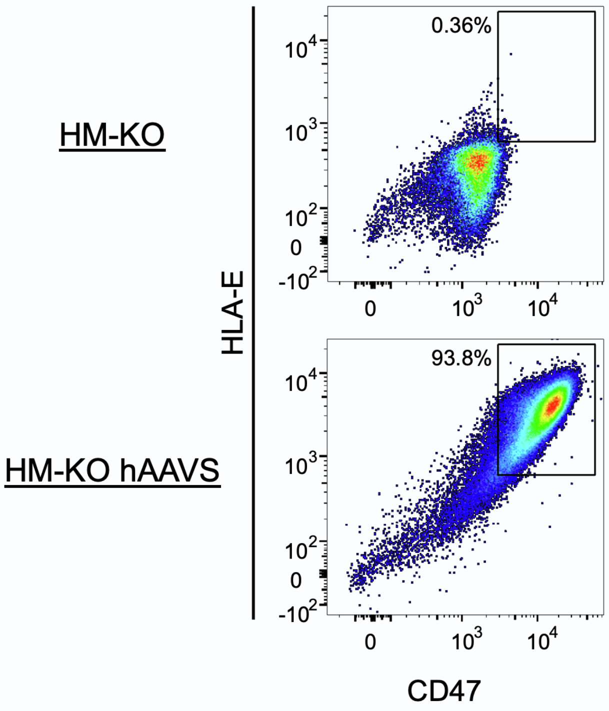
HM-KO
HM-KO hAAVS
HLA-E
0.36%
93.8%
CD47

Figure S2, related to Figure 3. Human immune evasion construct design and expression. (A) Two human AAVS constructs contain the inhibitory genes of interest. Each construct contains an A2UCOE insulator element, and expression is driven by the human elongation factor 1α (hEF1α) promoter. The immune evasion genes HLA-E SCT, hCD55, hCD46, HLA-G SCT, hCD47, and hCD59 are linked by 2A sequences. A suicide gene (iCasp9) is present in one construct, and both contain a neomycin resistance gene. The constructs have 5' and 3' AAVS1-targeting homology arms. (B) Flow cytometric analysis of human inhibitory protein expression on HM-KO cells, as well as HM-KO cells transfected with both human AAVS constructs (HM-KO hAAVS).

Figure S3

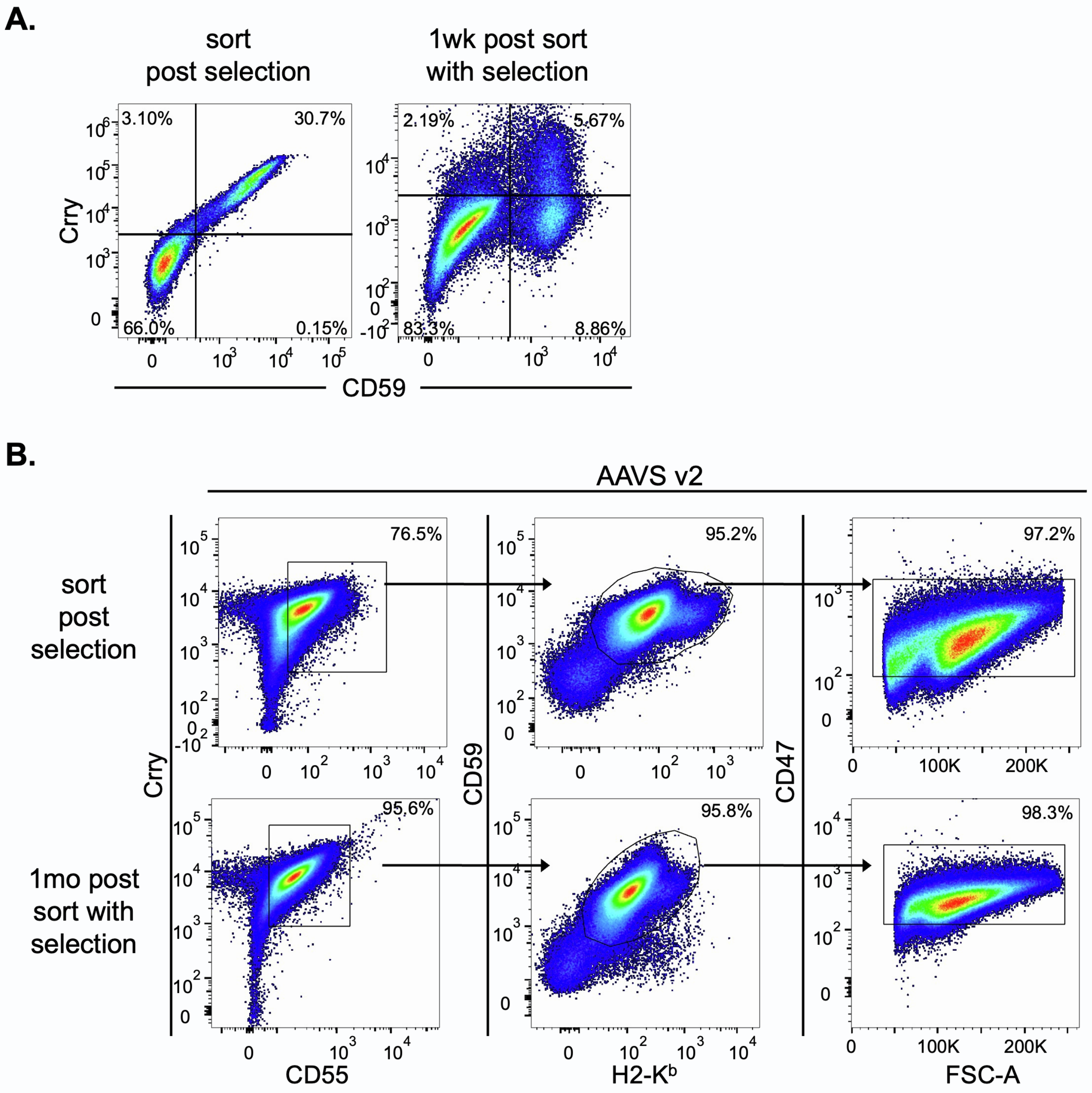

A.
sort
post selection
1wk post sort
with selection
3.10%
30.7%
66.0%
0.15%
2.19%
5.67%
83.3%
8.86%
Crry
CD59
B.
AAVS v2
sort
post
selection
1mo post
sort with
selection
76.5%
95.2%
97.2%
95.6%
95.8%
98.3%
Crry
CD59
CD47
CD55
H2-K^b
FSC-A
100K
200K

Figure S3, related to Figure 3. Modifying AAVS targeting site reduces silencing of expression. (A) Sample flow cytometry plots of immune evasion expression over time in HM-KO hESCs transfected with the original mouse AAVS constructs. Expression is shown following drug selection of transfected cells prior to sorting of $Crry^{+}$ $CD59^{+}$ double positive cells. Expression was then analyzed a week after bulk sorting and further drug selection. (B) Sample flow cytometry plots of immune evasion expression over time in HM-KO hESCS transfected with the redesigned AAVS constructs. The top row shows consecutive gating of transfected cells following drug selection. These cells were bulk sorted and kept on selection. Two months later expression was analyzed again as shown in the bottom row.

Figure S4

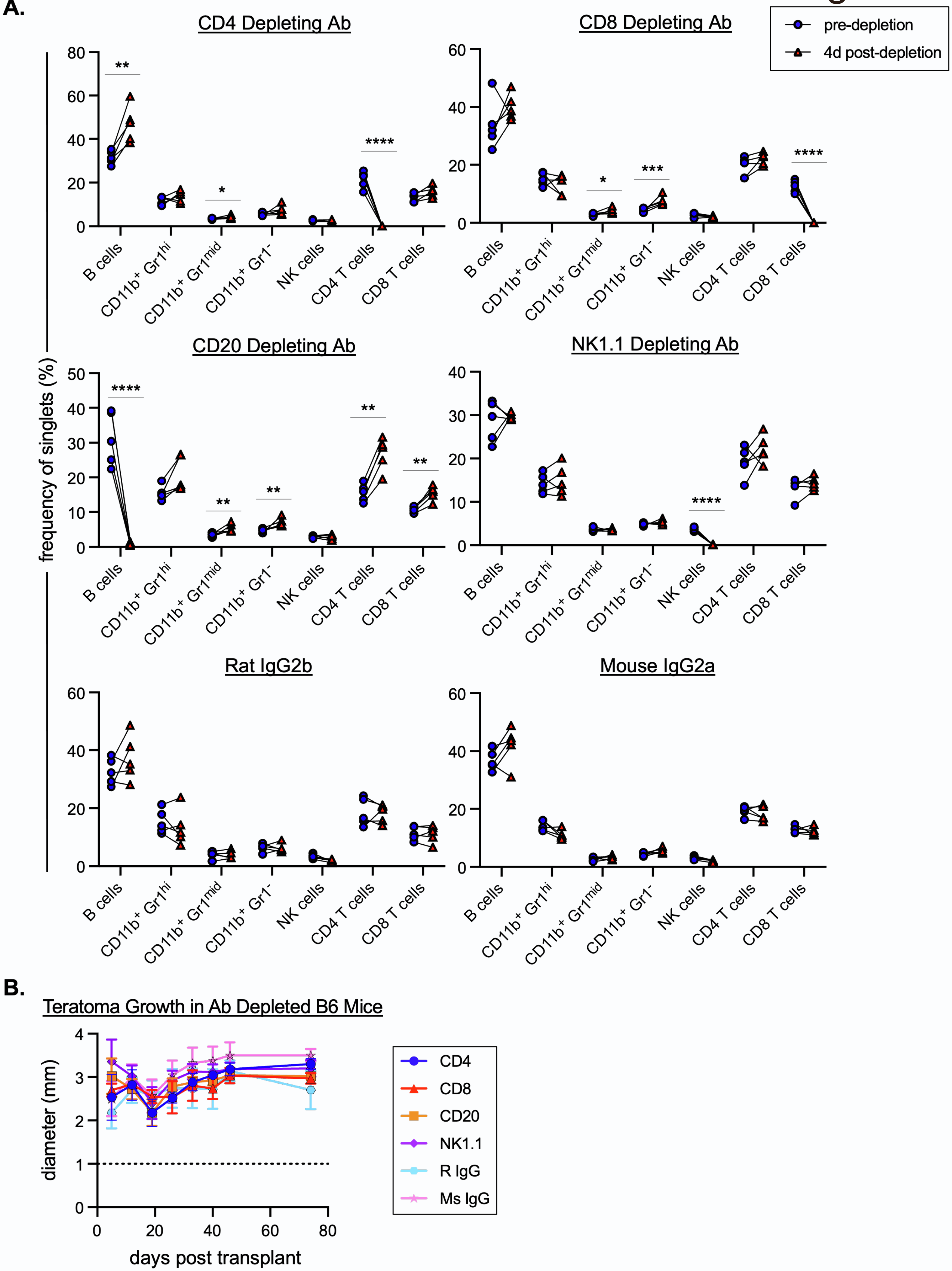

A.
CD4 Depleting Ab
CD8 Depleting Ab
CD20 Depleting Ab
NK1.1 Depleting Ab
Rat IgG2b
Mouse IgG2a
pre-depletion
4d post-depletion
frequency of singlets (%)
B cells
CD11b+ Gr1hi
CD11b+ Gr1mid
CD11b+ Gr1-
NK cells
CD4 T cells
CD8 T cells
B.
Teratoma Growth in Ab Depleted B6 Mice
diameter (mm)
days post transplant
CD4
CD8
CD20
NK1.1
R IgG
Ms IgG

Figure S4, related to Figure 4. Depletion of T cells, B cells, and NK cells does not enhance HM-KO $p53_{mut}$ lenti teratoma growth. (A) Confirmation of depletion of immune cells following antibody treatment. B6 mice were injected with CD4, CD8, CD20, or NK1.1 depleting antibodies or with control rat IgG2b or mouse IgG2a. Prior to injection of antibodies, mice were bled to determine the baseline levels of circulating immune cells. Four days following antibody injection, mice were bled to quantify the extent of depletion. Five wild-type mice are in each depleting antibody group, and pre-depletion and post-depletion frequencies are paired for each individual mouse. *p<0.05, **p<0.01, ***p<0.001, and ****p<0.0001 by Student's 2-tailed paired t-test. (B) Teratoma assay to assess growth of HM-KO $p53_{mut}$ lenti hESCs in B6 mice treated with depleting antibodies. Mice were injected with depleting antibodies 5d prior to the injection of HM-KO $p53_{mut}$ lenti hESCs. Throughout the assay, mice were injected weekly to maintain the depletion of each cell type. Mean values $\pm$ SEM are shown for each group of 5 mice. P values greater than 0.05 by 2-way ANOVA with post-hoc Tukey's multiple comparisons test are not depicted.

Figure S5

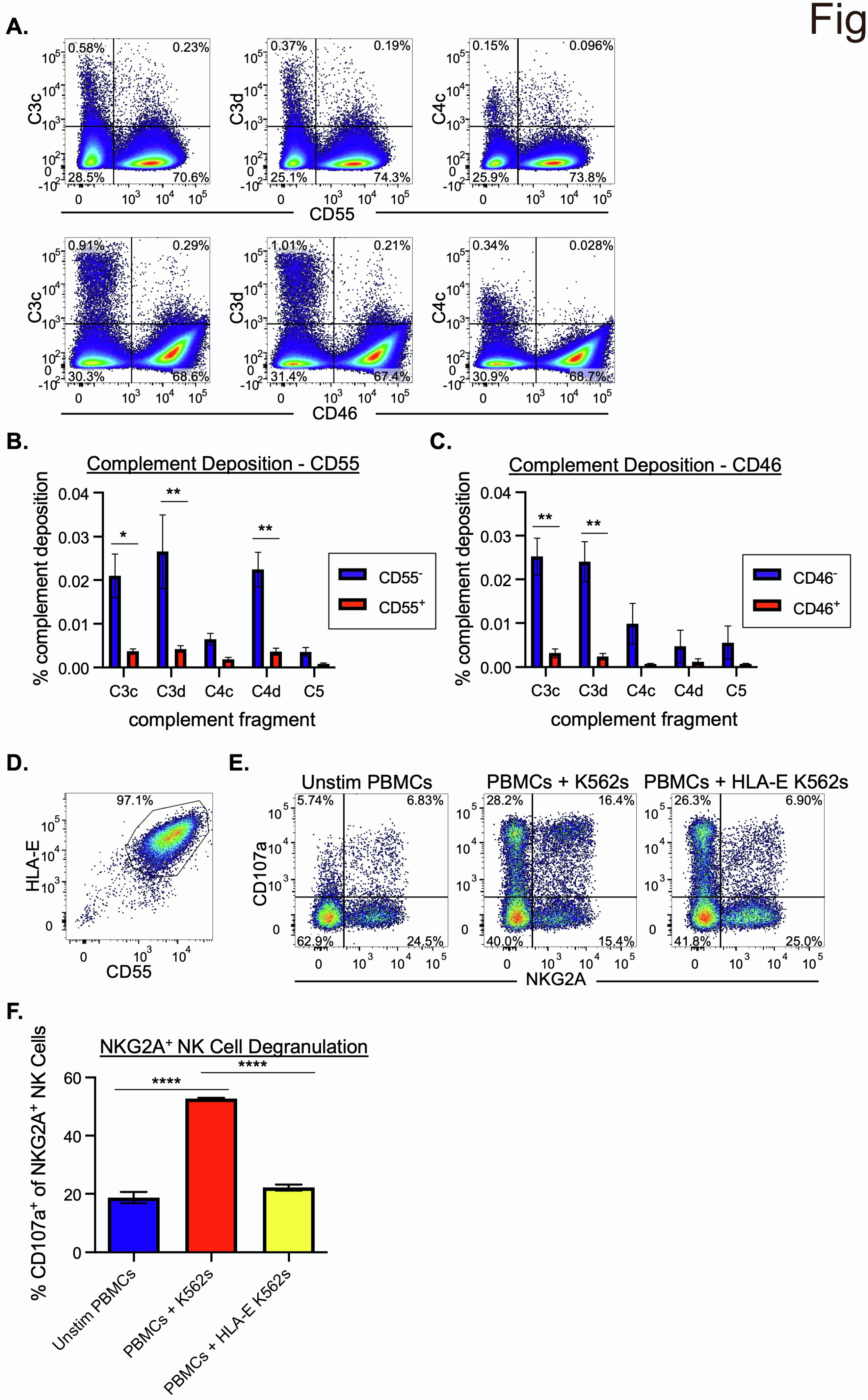

A.
C3c
C3d
C4c
CD55
CD46
0.58%
0.23%
28.5%
70.6%
0.37%
0.19%
25.1%
74.3%
0.15%
0.096%
25.9%
73.8%
0.91%
0.29%
30.3%
68.6%
1.01%
0.21%
31.4%
67.4%
0.34%
0.028%
30.9%
68.7%
B.
Complement Deposition - CD55
% complement deposition
complement fragment
C3c
C3d
C4c
C4d
C5
CD55⁻
CD55⁺
C.
Complement Deposition - CD46
CD46⁻
CD46⁺
D.
HLA-E
CD55
97.1%
E.
Unstim PBMCs
PBMCs + K562s
PBMCs + HLA-E K562s
CD107a
NKG2A
5.74%
6.83%
62.9%
24.5%
28.2%
16.4%
40.0%
15.4%
26.3%
6.90%
41.8%
25.0%
F.
NKG2A⁺ NK Cell Degranulation
% CD107a⁺ of NKG2A⁺ NK Cells
****
Unstim PBMCs
PBMCs + K562s
PBMCs + HLA-E K562s

Figure S5, related to Figure 4. Human CD55 and CD46 inhibit complement deposition, while HLA-E inhibits NKG2A$^+$ NK cell degranulation. (A) Representative flow cytometry plots of complement deposition on Chinese hamster ovary (CHO) cells with or without the expression of CD55 or CD46. CHO cells were transfected with the human AAVS constructs (Figure S2) containing either human CD55 (top row) or CD46 (bottom row). Cells were incubated with anti-CHO antibody followed by human C7-deficient serum and were stained for complement fragment deposition (C3c, C3d, C4c). (B-C) Quantification of complement deposition on cells expressing CD55 (B) or CD46 (C). Mean values $\pm$ SEM are shown for 3-7 replicates from 2 independent experiments. *$p<0.05$ and **$p<0.01$ by Student's 2-tailed t-test. (D) Flow cytometry plot of K562s transfected with the human AAVS construct containing HLA-E and hCD55. Cells were transfected, placed under selection, and sorted to obtain a pure population for downstream NK cell degranulation assays. (E) Representative flow cytometry plots of NK cell degranulation assays utilizing unstimulated PBMCs, PBMCs mixed with K562s, and PBMCs mixed with HLA-E-expressing K562s. Degranulation is measured by the expression of CD107a. Populations shown have been gated on CD56$^+$ NK cells. (F) NKG2A$^+$ NK cell degranulation assay. The frequency of CD107a-expressing cells of NKG2A$^+$ NK cells was analyzed from unstimulated PBMCs, PBMCs with K562s, and PBMCs with HLA-E$^+$ K562s. Mean values $\pm$ SEM are shown for 6 replicates from 2 independent experiments. ****$p<0.0001$ by Student's 2-tailed t-test.

Figure S6

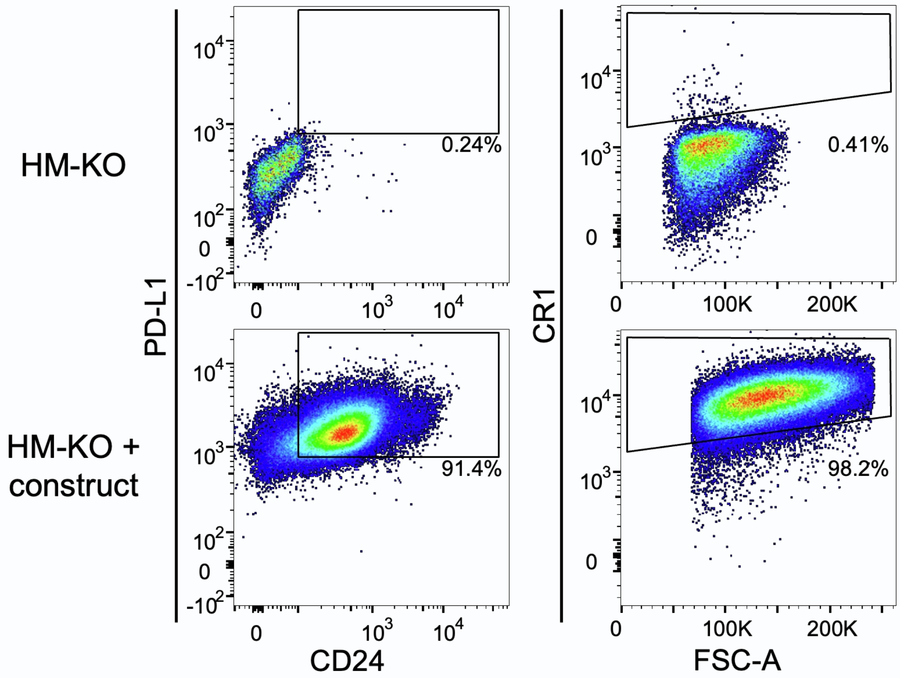
HM-KO
HM-KO +
construct
PD-L1
CD24
CR1
FSC-A
0.24%
91.4%
0.41%
98.2%
100K
200K

Figure S6, related to Figure 5. Expression of PD-L1, CD24, and CR1. Flow cytometric analyses of the expression of PD-L1, CD24, and CR1 on HM-KO cells and HM-KO cells transduced with PD-L1 and CD24 lentiviruses or transfected with a CR1 AAVS construct.

Table S1, related to Figure 1. Indel data for the 6 genes ablated to generate the HM-KO hESC line.

Targeted gene	Indel	Reads	Total reads (%)
β2M	-1	5032	52.9
	5	4454	46.9
TAP1	-2	1300	52.5
	1	1172	47.3
CD74	-10	787	48.9
	-8	756	47.0
CIITA	-2	456	51.4
	2	422	47.6
MICA	-1	1500	97.3
MICB	1	1064	98.0
MICA/B fusion	-13	460	95.8

A list of the insertions and deletions (indels) found for each gene ablated and the read count and frequency of each edited allele.

Table S2, related to Figures 1-7. hESC lines generated.

Name	Knocked out genes	Added genes (method)	Other notes
H1	-	-	wild-type
HLA-I/II-KO	*β2M*, *TAP1*, *CD74*, *CIITA*	-	*TP53* mutation
HM-KO $p53_{mut}$	*β2M*, *TAP1*, *CD74*, *CIITA*, *MICA*, *MICB*	-	*TP53* mutation
HM-KO	*β2M*, *TAP1*, *CD74*, *CIITA*, *MICA*, *MICB*	-	*TP53* mutation fixed
HM-KO $p53_{mut}$ lenti	*β2M*, *TAP1*, *CD74*, *CIITA*, *MICA*, *MICB*	*Crry*, *mCd55*, *mCd59*, *H2-K*^{b}, *mCd47* (lentiviruses)	*TP53* mutation fixed
HM-KO lenti	*β2M*, *TAP1*, *CD74*, *CIITA*, *MICA*, *MICB*	*Crry*, *mCd55*, *mCd59*, *H2-K*^{b}, *mCd47* (lentiviruses)	*TP53* mutation fixed
HM-KO AAVS	*β2M*, *TAP1*, *CD74*, *CIITA*, *MICA*, *MICB*	*Crry*, *mCd55*, *mCd59*, *H2-K*^{b}, *mCd47*, *Qa1* (AAVS constructs)	*TP53* mutation fixed
HM-KO comp	*β2M*, *TAP1*, *CD74*, *CIITA*, *MICA*, *MICB*	*Crry*, *mCd55*, *mCd59* (lentiviruses)	*TP53* mutation fixed
HM-KO NK	*β2M*, *TAP1*, *CD74*, *CIITA*, *MICA*, *MICB*	*H2-K*^{b}, *Qa1* (lentivirus)	*TP53* mutation fixed
HM-KO phago	*β2M*, *TAP1*, *CD74*, *CIITA*, *MICA*, *MICB*	*mCd47, mCD24, mPdl1* (lentiviruses)	*TP53* mutation fixed
HM-KO CR1	*β2M*, *TAP1*, *CD74*, *CIITA*, *MICA*, *MICB*	*mCr1* (AAVS construct)	*TP53* mutation fixed
HM-KO CR1 comp	*β2M*, *TAP1*, *CD74*, *CIITA*, *MICA*, *MICB*	*mCr1* (AAVS construct), *mCd59* (lentivirus)	*TP53* mutation fixed
HM-KO NK and comp	*β2M*, *TAP1*, *CD74*, *CIITA*, *MICA*, *MICB*	*Crry*, *mCd55*, *mCd59*, *H2-K*^{b}, *Qa1* (lentiviruses)	*TP53* mutation fixed
HM-KO CR1 NK and comp	*β2M*, *TAP1*, *CD74*, *CIITA*, *MICA*, *MICB*	*mCr1* (AAVS construct), *mCd59*, *H2-K*^{b}, *Qa1* (lentiviruses)	*TP53* mutation fixed

Modifications and nomenclature of edited hESC lines.

Table S3, related to Figure 2. Donor chimerism of huNSG-W41 mice.

	hCD45⁺ cells (% of single cells)	T cells (% of hCD45⁺ cells)	B cells (% of hCD45⁺ cells)	Myeloid cells (% of hCD45⁺ cells)
blood	0.46	0	78.7	12.9
	3.4	0.093	93.9	3.02
	1.48	0.2	83.5	6.27
	7.99	0.04	90	5.72
	6.44	0.57	91.1	4.31
	1.51	0.062	90.2	4.64
	0.56	0	91.7	1.39
	3.41	0.14	92.2	3.76
	2.31	0.26	91.2	3.04
	10.2	0.46	90.9	3.96
	0.18	0	88.3	5.37
	14.8	0.1	94.3	2.19
	1.13	0.33	92.6	0
	0.54	0.41	78.1	4.96
	0.93	0.69	85.4	3.47
	2.66	0.21	92	1.92
	0.95	3.66	86.6	6.1
	0.2	1.74	78	7.42
	2.8	0.15	85.2	7.42
	0.82	1.98	90.1	1.98
	0.32	0	74.5	14.5
	1.16	0.021	87.4	6.32
	5.47	0.018	91	2.38
	2.27	0.026	84.1	5.83
	2.95	0.01	92	2.81
	0.15	0	60.4	25
	0.44	2.11	64.1	23.2
	4.43	0.2	89.5	4.77
	7.76	0.31	89.6	4.66
	9.18	0.1	91.7	3.32
	8.72	49.2	37.8	3.83
	0.94	4.47	76.3	4.81
	1.66	0.26	95.2	0.74
	3.19	0.48	73.4	18.1
	1.58	0.52	83.5	8.26
	0.69	1.25	96.2	0
	5.07	0.083	93.5	2.19
	12.4	0.24	91.2	3.37
	3.99	0.049	86.9	5.27
	1	0.14	88.3	4.99
	2.65	0.011	87	4.04

	7.12	0.21	84.8	6.05
	0.04	0.021	87.9	6.09
	0.69	0.34	82.4	4.41
	5	0.088	88.8	1.81
	3.87	0.027	83.3	8.17
	4.87	0.097	82.3	8.97
	2.73	0.032	82.9	9.35
	2.58	0.29	75.7	13
	1.72	0.23	71.9	14
	5.44	0.041	82.2	8.29
	0.59	0	75.6	14.7
	1.17	0.23	76.2	15.4
	0.64	0.01	70.3	11
	1.04	0.61	68.2	12.4
	2.15	0.14	80	10.8
	4.48	0.28	70.9	15.6
	6.85	0.072	90	3.36
	0.21	0	88.5	2.27
	5	0.11	85.3	4.27
	6.01	0.28	86.3	5.91
	1.52	0.15	89.9	4.84
	3.07	0.091	84.1	6.49
spleen	44	45.4	43.7	2.18
	44.6	78.6	18.4	2.83
	67.4	2.91	91.1	1.82
	52.3	34.5	52.6	2.01
	56.1	57.2	30.7	2.44

Human chimerism ($hCD45^+$) of cells in the blood or spleen of huNSG-W41 mice 2+ months post-transplantation of human cord blood $CD34^+$ cells. The human cells were further divided into T cells ($CD3^+$), B cells ($CD19^+$), and myeloid cells ($CD13^+$). An example flow cytometry plot of human chimerism and cell subsets, as well as data pooled into bar graphs, are shown in Figures 2A and B.

Table S4, related to Figure 3. Immune evasion proteins.

<table>
<tr><th></th><th>Human proteins</th><th>Mouse proteins</th><th>Inhibits</th></tr>
<tr><td rowspan="7">In AAVS Constructs</td><td>CD46 (MCP)</td><td>Crry</td><td>Complement/C3b and C4b</td></tr>
<tr><td colspan="2">CD55 (DAF)</td><td>Complement/C3 and C5 convertases</td></tr>
<tr><td colspan="2">CD59</td><td>Complement/membrane attack complex</td></tr>
<tr><td>HLA-E</td><td>Qa1</td><td>NKG2A$^+$ NK cells</td></tr>
<tr><td>HLA-G</td><td>-</td><td>ILT2/KIR2DL4$^+$ NK cells</td></tr>
<tr><td>-</td><td>H2-K^b</td><td>Ly49C$^+$ NK cells</td></tr>
<tr><td colspan="2">CD47</td><td>Phagocytes (SIRPα)</td></tr>
<tr><td></td><td colspan="2">CD24</td><td>Phagocytes (Siglec-10)</td></tr>
<tr><td></td><td colspan="2">PD-L1</td><td>Phagocytes (PD-1), antigen presentation, T cell activation</td></tr>
<tr><td></td><td colspan="2">CR1</td><td>Complement/C3b and C4b, C3 and C5 convertases</td></tr>
</table>

Human and mouse inhibitory factors in immune evasion constructs and the cell type or pathway that they inhibit.

Table S5, related to experimental procedures. Gene targeting to generate the HM-KO hESC line.

Targeted gene	gRNA spacer (5' to 3')	Forward primer (5' to 3')	Reverse primer (5' to 3')
β2M	GGCCACGGAGCGAGACAUCU	CGCCGATGTACAGACAGCAA	TTGGAGAAGGGAAGTCACGG
TAP1	GUGAGGCGGCCCGUAAAGAA	TTTCTGCTGATGCCCTCCAG	GGATCAGGGTGTGTTCAGGG
CD74	UGUUGGAGAUAAGGUCGCGC	GACTTTAAGAGGCGAGCCGG	GGCGGTGTGATGCAAGGAAA
CIITA	AUGGAGUUGGGGCCCCUAGA	GCGCTTTTATTCACTCCTCTCATCCCCA	GCGGGGCAGGGCTGGGAGATCATC
MICA	GGAGAAGUUUGUUGGCCUGG	TGCATGCAAAGTATGTCCCA	-
MICB	GUAAUGCAUAACGGUGUGAC	-	TTTGATGCTGGGTCAAGGCA
MICA/B fusion	CAAGAAAUUCCUGACGUUCA	CCTGTCTCTTCCCACTGGATCT	GGTCCTCTACTTGCCCTGATTAC

gRNA spacers and primers used for gene targeting of H1 hESCs to generate HM-KO hESCs.

Supplemental experimental procedures

TP53 mutation correction

TP53 was sequenced with the Accel-Amplicon Comprehensive *TP53* Panel (Swift Biosciences) according to the manufacturer's protocol. To correct the *TP53* mutation, HM-KO hESCs were nucleofected with a complex of Cas9 protein bound to a gRNA targeting 5' GCATGGGCGGCATGAACCAGNGG 3' along with a correction ssODN 5' CCTGGAGTCTTCCAGTGTGATGATGGTGAGGATGGGCCTCCTGTTCATGCCGCCCATGCAGGAACT GTTACACATGTAGTTG 3' and GFP plasmid. Cells were single cell-sorted and screened for correction using Miseq with forward primer 5' AGATCACGCCACTGCACTCCAGCCT 3' and reverse primer 5' CGCCGGGGATGTGATGAGAGGTGGA 3'. Cells were expanded and frozen down within the first 5 passages, and cells from these early passages were used to generate subsequent lines.

Whole exome sequencing

DNA was isolated using Genomic DNA Mini Kit (IBI Scientific). Whole exome sequencing was performed by the University of Chicago Genomics Facility. Sequencing data was processed using the BWA pipeline from the Broad Institute[1] and then by the Ensembl Variant Effect Predictor[2]. Concerning mutations were identified using the Human Clinical Variation Database[3]. Sequencing was performed in 2018, and cells frozen down within 5 passages were used as parental stocks.

Flow cytometry antibodies

The following human antibodies were purchased from BioLegend: β2-microglobulin (2M2) – APC; TruStain FcX; HLA-A, B, C (W6/32) – APC/Cy7; HLA-E (3D12) – PE; HLA-G (87G) – APC, PE/Dazzle 594; MICA/MICB (6D4) – PE, PerCP/Cy5.5; HLA-DR (L243) – Brilliant Violet 605, Brilliant Violet 650; CD7 (CD7-6B7) – PE; CD34 (581) – PE/Cy7, APC/Cy7; CD4 (A16A1) – PE; CD4 (OKT4) – Brilliant Violet 421, APC; CD8 (SK1) – APC/Cy7; CD14 (M5E2) – PE/Cy7; CD13 (WM15) – PE/Cy7; CD43 (CD43-10G7) – APC, PE/Cy7; CD45RA (HI100) – Alexa Fluor 700; CD45 (2D1) – APC; CD73 (AD2) – PE; CD1c (L161) – Brilliant Violet 510; CD3 (OKT3) – PerCP/Cy5.5; CD10 (HI10a) – PerCP/Cy5.5; CD11b (ICRF44) – Brilliant Violet 650; CD19 (HIB19) – Brilliant Violet 421; CD19 (SJ25C1) – Brilliant Violet 510; CD33 (WM53) – PerCP/Cy5.5; CD34 (561) – FITC; CD38 (HIT2) – PE/Cy7, biotin; CD46 (TRA-2-10) – APC/Cy7, PE; CD47 (CC2C6) – PE/Cy7; CD49f (GoH3) – Brilliant Violet 421; CD49d (9F10) – PE/Cy5; CD55 (JS11) – APC; CD56 (5.1H11) – APC; CD56 (HCD56) – PE/Cy7, PE/Dazzle 594; CD59 (H19) – FITC; CD85j (GHI/75) – biotin; CD90 (5E10) – APC; CD107a (H4A3) – Brilliant Violet 421; CD141 (M80) – PE; CD158d (mAb 33 (33)) – PE; CD184 (12G5) – Brilliant Violet 421, PE/Cy7; CD135 (BV10A4H2) – biotin; TCR γ/d (B1) -PE; CD11c (Bu15) – Alexa Fluor 700; Nestin (10C2) – PE. The following human antibodies were purchased from BD: CD11b (ICRF44) – Alexa Fluor 488; CD16 (3G8) – APC-Cy7; CD33 (HIM3-4) – FITC; CD46 (E4.3) – FITC; CD59 (p282 (H19)) – PE. Human HLA-ABC (W6/32) – PE was purchased from Invitrogen. Human CD159a (REA110) – FITC was purchased from Miltenyi Biotec. The following mouse antibodies were purchased from BioLegend: CD3 (145-2C11) – Brilliant Violet 510, PerCP/Cy5.5; CD4 (RM4-5) – Brilliant Violet 605; CD8 (53-6.7) – Alexa Fluor 700; CD19 (6D5) – Brilliant Violet 421; CD45 (30-F11) – Alexa Fluor 488; B220 (RA3-6B2) – FITC; NK-1.1 (S17016D) – PE; Ly-6G/Ly-6C (RB6-8C5) – APC; CD47 (miap301) – FITC, PE, Brilliant Violet 421, APC/Cy7, PE/Dazzle 594; CD55 (RIKO-3) – PE, PE/Cy7; CD59 (mCD59.3) – PE; CD274 (10F.9G2) – PE, Brilliant Violet 605, Brilliant Violet 650; H-2Kb (AF6-88.5) – Alexa Fluor 647, FITC, Brilliant Violet 421; CD29 (HMβ1-1) – APC/Cyanine7, Alexa Fluor 488; Ig light chain κ (RMK-45) – FITC; CD21/35 (7E9) – APC; CD24 (M1/69) – Alexa Fluor 700, Brilliant Violet 510, Pacific Blue. The following mouse antibodies were purchased from BD: CD11b (M1/70) – PE/Cy7; Crry/p65 (1F2) – biotin, BV786; CD24 (M1/69) – PE/Cy7. The following mouse antibodies were purchased from Miltenyi Biotec: CD55 – biotin; Qa-1b (6A8.6F10.1A6) – APC; Qa-1b – biotin. Mouse IgG – UNLB was purchased from Southern Biotech. Streptavidin – BV605, BV421, PE-Cy7 were purchased from BD. The following transcription factor antibodies were purchased from Invitrogen: brachyury recombinant rabbit monoclonal antibody (JE44-11), SOX17 rabbit polyclonal antibody, and PAX6 rabbit polyclonal antibody. Goat anti-rabbit IgG superclonal recombinant secondary antibody in Alexa Fluor 647 from Invitrogen was used as a secondary stain for transcription factors. DAPI and propidium iodide were purchased from Sigma-Aldrich. Zombie Violet Fixable Viability Kit was purchased from BioLegend and used per the manufacturer's instructions.

DC-like cell differentiations

hESCs were differentiated into hematopoietic progenitors using either an embryoid body culture[4] or the STEMdiff Hematopoietic Kit (STEMCELL Technologies, cat. no. 05310) per the manufacturer's instructions. Hematopoietic progenitors were collected then cultured in flasks coated with 20mg/mL poly-HEMA (Sigma-Aldrich, cat. no. P3932-10G) in α-MEM with 10% fetal bovine serum (FBS), Glutamax, penicillin/streptomycin, and 100ng/mL hGM-CSF (PeproTech, cat. no. 300-03) for 8-10d with half medium changes every 4d[5]. Cells were collected and spun over 20% Percoll (GE Healthcare). The cells at the interface were collected and cultured in poly-HEMA-coated flasks with StemSpan SFEM (STEMCELL Technologies, cat. no. 09650) supplemented with lipid mixture 1 (Sigma-Aldrich, cat. no. L0288-100M), 100ng/mL hGM-CSF (Peprotech, cat. no. 300-03), and 100ng/mL hIL-4 (PeproTech, cat. no. 200-04) for 7-9d with half medium changes every 4d. Cells were then collected and cultured in poly-HEMA-coated flasks in StemSpan SFEM with lipid mixture 1 and 400ng/mL A23187 calcium ionophore (Sigma-Aldrich, cat. no. C7522-5MG) for 2d. Human PBMCs were used as a control following culture in RPMI with 10% FBS, 2-mercaptoethanol, penicillin/streptomycin, 100ng/mL hGM-CSF, and 100ng/mL hIL-4 for 7-10d.

Blood and spleen processing of humanized mice
At least 2mo post-transplant, mice were bled to confirm human chimerism. Peripheral blood was collected in 10mM EDTA/PBS via tail venipuncture of warmed mice. Red blood cells (RBCs) were lysed with 0.15M NH_4Cl, 10mM $KHCO_3$, 0.1mM EDTA, pH 7.2 solution (ACK). For splenic chimerism, spleens were harvested and dissociated with frosted glass microscope slides. RBCs were lysed with ACK. Cells were filtered through 70μm nylon mesh prior to staining.

Lentivirus production
Lenti-X 293T cells (Takara Bio USA) were cultured at 37°C with 5% CO_2 in DMEM with 10% FBS, nonessential amino acids, Glutamax, sodium pyruvate, and penicillin/streptomycin. Cells were transfected at ~60% confluency in 10cm^2 tissue culture plates using 30μL GeneJuice Transfection Reagent (Sigma-Aldrich, cat. no. 70967) with 5μg lentiviral vector, 3.25μg psPax2 (Addgene 12260), and 1.75μg VSV.G (Addgene 12259). Medium was changed 6-8hr post-transfection, and viral supernatant was harvested 48 and 72hr later. 12mL viral supernatant was mixed with 3mL 25% polyethylene glycol 8000 (Sigma-Aldrich) in PBS and incubated overnight at 4°C. This mixture was centrifuged at 3,000 x g for 20 min, supernatant was discarded, and the pellet was resuspended in 100μL PBS. Aliquoted lentivirus was stored at -80°C.

Antibody depletion
Mice were bled (as described in the blood and spleen processing section above) prior to the first injection of depleting antibodies. Mice were injected intraperitoneally with 250µg of one of the following depleting antibodies: mouse IgG2a (clone C1.18.4; BioXCell), rat IgG2b (BioXCell), CD8 (clone YTS169.4; BioXCell), CD4 (clone GK1.5; BioXCell), CD20 (clone SA271G2; BioLegend), or NK1.1 (clone PK136; BioXCell). Four days after injection, mice were bled to confirm depletion. hESCs were transplanted into these mice 5d after the initial depleting antibody injection. Depleting antibodies were administered weekly throughout the teratoma assay.

Chinese hamster ovary cell culture
Chinese hamster ovary (CHO) cells were cultured in DMEM with 10% FBS and penicillin/streptomycin. Cells were maintained at 37°C with 5% CO_2. Human AAVS constructs were transfected into CHO cells using the Gene Pulser MXcell Electroporation System (Bio-Rad). Transfected cells were selected for with 10µg/mL puromycin or 1mg/mL neomycin. Cells expressing the immune evasion proteins were also purified via FACS.

Complement deposition assay
CHO cells were incubated with 1µg/mL anti-CHO antibody (Cygnus Technologies, cat. no. C0016-PA) for 30min at 4°C. Cells were washed with either GVB++ or GVB^o + MgEGTA (Complement Technology, cat. no. B102 and B103, respectively) for the classical or alternative pathway, respectively. Cells were resuspended in 10% C7-deficient serum (Sigma-Aldrich, cat. no. C1413-1ML) in the appropriate GVB buffer and incubated at 37°C for 45min with shaking. Cells were washed with 1% FBS/PBS then stained with mouse anti-human C3c, C3d, C4c, C4d, or C5 antibody (Quidel, cat. no. A205, A207, A211, A213, and A217) for 30min at 4°C. Cells were washed with 1% FBS/PBS then stained with PE/Cy7-conjugated rat

anti-mouse Igκ light chain antibody (BD) for 30min at 4ºC. Cells were washed and blocked with unlabeled mouse IgG (Southern Biotech) prior to subsequent staining and analysis.

K562 cell culture
K562s were cultured in DMEM with 10% FBS, Glutamax, nonessential amino acids, sodium pyruvate, and penicillin/streptomycin. Cultures were maintained at 37ºC with 5% CO_2. Human AAVS constructs were transfected into K562s using the Gene Pulser MXcell Electroporation System (Bio-Rad). Transfected cells were selected for with 10µg/mL puromycin or 1mg/mL neomycin. Cells expressing the immune evasion proteins were also purified via FACS.

NK cell CD107a degranulation assay
PBMCs were plated in RPMI with 10% FBS, Glutamax, nonessential amino acids, sodium pyruvate, HEPES, and penicillin/streptomycin supplemented with 1ng/mL hIL-15 (PeproTech, cat. no. 200-15) and cultured overnight at 37ºC with 5% CO_2. PBMCs were either cultured alone (unstimulated) or at a 10:1 ratio with K562s. Cells were stained with CD107a for 1hr at 37ºC. GolgiStop/Plug (BD, cat. no. 554724 and 555029) was then added per manufacturer's instructions and cells were incubated for 5hr at 37ºC. Cells were washed and stained for analysis.

Immunohistochemistry (IHC)
Teratomas were harvested and fixed in 4% paraformaldehyde solution (Santa Cruz Biotechnology, Inc.) for 24hr at room temperature then washed with PBS 3 times and stored in PBS at 4ºC. Samples were cryoprotected with sucrose (Sigma-Aldrich), cryopreserved in Tissue-Tek O.C.T. compound (Sakura), and sectioned at 5µm with a Leica CM3050 S cryostat. Sections were incubated with 0.1% triton X-100 (Sigma-Aldrich) and 5% donkey serum (Jackson ImmunoResearch) in PBS. Sections were incubated overnight with the following antibodies unconjugated goat anti-mouse/rat CD47 N-terminal IgV-like extracellular domain (R&D Systems), biotin-conjugated anti-mouse CD55 REAfinity (clone REA300, Miltenyi Biotec), unconjugated rabbit anti-mouse CD59a (clone 108, SinoBiological), and AF594-conjugated anti-human mitochondria (clone 113-1, Novus Biologicals) followed by a 1hr incubation with Alexa Fluor 647-conjugated AffiniPure donkey anti-goat IgG antibody (Jackson ImmunoResearch), Alexa Fluor 647-conjugated streptavidin (Jackson ImmunoResearch), or Alexa Fluor Plus 555-conjugated donkey anti-rabbit IgG antibody (Invitrogen). Slides were mounted with ProLong Gold Antifade Mountant with DAPI (Invitrogen) and imaged on a Zeiss LSM 800 confocal laser scanning microscope. Images were processed and analyzed with Fiji (ImageJ).

Supplemental references

[1]McKenna, A., Hanna, M., Banks, E., Sivachenko, A., Cibulskis, K., Kernytsky, A., Garimella, K., Altshuler, D., Gabriel, S., Daly, M., et al. (2010). The Genome Analysis Toolkit: A MapReduce framework for analyzing next-generation DNA sequencing data. Genome Res. *20*, 1297–1303. https://doi.org/10.1101/gr.107524.110.

[2]McLaren, W., Gil, L., Hunt, S.E., Riat, H.S., Ritchie, G.R.S., Thormann, A., Flicek, P., and Cunningham, F. (2016). The Ensembl Variant Effect Predictor. Genome Biol. *17*, 122. https://doi.org/10.1186/s13059-016-0974-4.

[3]Landrum, M.J., Lee, J.M., Riley, G.R., Jang, W., Rubinstein, W.S., Church, D.M., and Maglott, D.R. (2014). ClinVar: public archive of relationships among sequence variation and human phenotype. Nucleic Acids Res. *42*, 980-985. https://doi.org/10.1093/nar/gkt1113.

[4]Dege, C., and Sturgeon, C.M. (2017). Directed Differentiation of Primitive and Definitive Hematopoietic Progenitors from Human Pluripotent Stem Cells. J. Vis. Exp. *129*, 1-9. https://doi.org/10.3791/55196.

[5]Slukvin, I.I., Vodyanik, M.A., Thomson, J.A., Gumenyuk, M.E., and Choi, K.-D. (2006). Directed differentiation of human embryonic stem cells into functional dendritic cells through the myeloid pathway. J. Immunol. *176*, 2924–2932. https://doi.org/10.4049/jimmunol.176.5.2924.
